# Exploration of Turkish ICU Nurses’ care experiences during the COVID-19 pandemic: A qualitative study

**DOI:** 10.1590/1980-220X-REEUSP-2025-0274en

**Published:** 2026-01-30

**Authors:** Demet Acar, Araz Askeroğlu

**Affiliations:** 1 Çanakkale Onsekiz Mart University Hospital, Department of General Surgery, Çanakkale, Turkey.; 2 Çanakkale Onsekiz Mart University, Faculty of Health Sciences, Department of Surgical Nursing, Çanakkale, Turkey.

**Keywords:** COVID-19, Intensive Care Unites, Nursing, Patient Safety, COVID-19, Unidades de Terapia Intensiva, Enfermagem, Segurança do Paciente

## Abstract

**Objetive:**

This phenomenological study aims to describe the care experiences of Turkish ICU nurses during the COVID-19 pandemic.

**Method:**

Data were collected through semi-structured interviews with 12 ICU nurses. This study was conducted in public hospitals, which were the main centers of the pandemic in Türkiye. Data were analyzed by descriptive, comparative and relational analysis methods.

**Results:**

A total of 851 primitive codes were identified. Four themes, 12 categories, 47 codes, and 13 sub-codes were determined: (a) Categories of the theme “Holistic Care Approach”: barriers, communication, and spiritual care. (b) Categories of the theme “Process Management”: precautions, challenges, adaptation, and treatment processes. (c) Categories of the theme “Physical-Psychological Effects”: physical effects and psychological effects. (d) Categories of the theme “Ethical Dilemma”: ethical behaviors, unethical behaviors, and working principles. In the relational analysis, participants expressed their opinions on challenges, as well as on precautions, workload, duration of care, and health deterioration.

**Conclusion:**

Participants reported both positive and negative care experiences. Negative experiences can adversely impact patient safety, whereas positive experiences serve as a source of hope and motivation, encouraging nurses to continue providing holistic care.

## RESUMO

**Objetivo:**

Este estudo fenomenológico teve como objetivo descrever as experiências de cuidado de enfermeiros(as) de UTI turcos(as) durante a pandemia de COVID-19.

**Método:**

Os dados foram coletados por meio de entrevistas semiestruturadas com 12 enfermeiros(as) de UTI. Este estudo foi realizado em hospitais públicos, que foram os principais centros da pandemia na Turquia. Os dados foram analisados por métodos descritivos, comparativos e de análise relacional.

**Resultados:**

Um total de 851 códigos primários foram identificados. Quatro temas, 12 categorias, 47 códigos e 13 subcódigos foram determinados: (a) Categorias do tema “Abordagem de Cuidado Holístico”: barreiras, comunicação e cuidado espiritual. (b) Categorias do tema “Gestão do Processo”: precauções, desafios, adaptação e processos de tratamento. (c) Categorias do tema “Efeitos Físico-Psicológicos”: efeitos físicos e efeitos psicológicos. (d) Categorias do tema “Dilema Ético”: comportamentos éticos, comportamentos não éticos e princípios de trabalho. Na análise relacional, os participantes expressaram suas opiniões sobre desafios, bem como sobre precauções, carga de trabalho, duração do cuidado e deterioração da saúde.

**Conclusão:**

Os participantes relataram experiências de cuidado tanto positivas quanto negativas. Experiências negativas podem afetar negativamente a segurança do paciente, enquanto experiências positivas servem como fonte de esperança e motivação, incentivando os(as) enfermeiros(as) a continuar prestando cuidados holísticos.

## INTRODUCTION

In March 2020 the World Health Organization (WHO) highlighted that healthcare professionals involved in the fight against COVID-19 faced numerous risks, including pathogen exposure, excessive workloads, fatigue, professional burnout, and physical violance as well as psychological violence. Thesudden and rapidly increasing healthcare demand caused by the COVID-19 pandemic significantly strained global healthcare systems^(1,2)^. The literature indicates that nurses caring for patients diagnosed with COVID-19 often encounter significant challenges, such as feelings of being overwhelmed and exhausted due to workloads and the continuous requirement to wear Personal Protective Equipment (PPE). Additionally, nurses expressed concerns regarding the inadequacy of PPE, the risk of contracting the virus, and the potential to transmit the disease to their families. These issues are further compounded by worries about their ability to provide adequate and necessary care to patients, along with physical and psychological disorders such as excessive sweating, feelings of fatigue, anxiety, and helplessness^(1,2,3,4,5,6)^. Studies have shown that while wearing PPE was mandatory for those working in COVID-19 ICUs, it caused stress, anxiety, and various physical and psychological challenges^(7,8)^. Furthermore, Intensive Care Unit (ICU) nurses faced significant challenges while working in COVID-19 units, including the need to postpone basic needs and workloads and other difficulties^(3,5,9,10)^. Although nurses in COVID-19 ICU are the first point of contact with patients, factors such as workload, staff shortages, risk of infection, and the use of PPE have been identified as challenges that hinder nurses from providing holistic care to patients^(5,11,12,13)^. Furthermore, nurses, serving as the sole link between patients and their families, play a critical role in addressing the emotional needs of patients^(14)^. The literature highlights that the spiritual support provided by nurses is crucial for COVID-19 patients^(2,15)^.

COVID-19 placed healthcare workers, particularly nurses, under intense stress in Türkiye, as it did worldwide. The number of intensive care beds in Türkiye increased during the COVID-19 pandemic, leading ICU nurses to work under extremely harsh conditions. Concerns were raised regarding nurses being at heightened risk of contracting COVID-19, with the rising number of patients and inadequate staffing contributing to workloads, fatigue, team communication conflicts, and disruptions in the quality of care provided^(16)^.

Studies examining the experiences of ICU nurses during the COVID-19 pandemic highlight significant physical, psychological, and professional challenges they faced. A study revealed that ICU nurses faced immense burdens due to the lack of evidence-based treatments, poor patient prognoses, inadequate leadership support, and insufficient community backing. The study emphasized the neet for further research to better understand nurses’ experiences and to develop resources that support their well-being^(17)^. A review study assessing the impact of the pandemic on ICU nurses idendified several key areas of influence, including increased workload, changes in staffing organization, challenges experienced vyredeployed personnel, perceptions of patient safety and care quality, and effects on nurses’ health^(18)^. Similarly, a qualitative study found that ICU nurses experienced intense psychological and physical effects, while also navigating societal shifts and varying public perceptions of the pandemic^(19)^. In Iran, research examining the challenges ICU nurses encountered while caring for COVID-19 patients identified psychological distress, physical exhaustion, organizational inefficiencies and uncertainty regarding the disease as major concerns. The study emphasized the importance of understanding these challenges to enable healthcare authorities to implement appropriate interventions, improve healthcare facilities, support the workforce, and provide evidence-based information and psychological support^(6)^. A qualitative study conducted in Spain explored ICU nurses’ experiences and perceptions during the pandemic, identifying both strengths and weaknesses in the healthcare system. The study found that fear and isolation influenced nursing care, making it difficult to maintain humanized healthcare practices. It also underscored the need for optimized resource management, psychological support, adequate training, and the development of high- quality protocols for future emergencies^(3)^. A scoping review investigating burnout among ICU nurses identified high levels of burnout, with contributing factors including lack of equipment, social stigma, increased workload and fear of contagion. However, social support and professional recognition were found to be significant protective factors against burnout^(20)^. A separate study in Spain reported that critical care and emergency nurses faced excessive workloads, high patient-nurse ratios, and emotional exhaustion throughout the pandemic^(9)^. Further research indicated that ICU nurses faced challenges in collaborating with new colleagues, maintaining existing workplace relationships, and lacking institutional recognition, which contributed to emotional and physical exhaustion^(21)^. In Iran, additional challenges reported by ICU nurses included a disconnect from holistic nursing, organizational inefficiencies, job burnout, and evolving workplace Dynamics^(22)^. Despite these challenges, some studies highlighted ICU nurses’ resilience, adaptability, and strong leadership in managing patient care during the crisis^(23,24)^. However, patient safety and care quality were often compromised, leading to ethical stress and adverse effects on nurses’ physical and psychosocial well-being^(25)^. A study results emphasized the critical role of teamwork and colleague support in helping ICU nurses cope with extreme conditions during the pandemic^(26)^. According to the results of the literature review the COVID-19 pandemic placed substantial strain on ICU nurses, affecting their workload, psychological well-being, and the quality of patient care. The study aimed to describe the care experiences of ICU nurses during the COVID-19 pandemic in Türkiye.

## DESIGN AND METHODS

### Setting and Sample

In this qualitative, phenomenological- descriptive, multi- center study data was collected through Zoom Cloud Meeting platform. This study was conducted in public hospitals, which were the main centers of the pandemic in Türkiye between April 2021–September 2021. The study sample consisted of ICU nurses working during the COVID-19 pandemic, selected using the snowball sampling method. ICU nurses were approached via social media platforms. Additionally, nurses from various hospitals were invited to join through referrals from those who hadalready agreed to participate. The interviews were conducted by the first author in Turkish, following an introduction of herself and the study objectives. Nurses who actively working in ICUs during the COVID-19 pandemic, and who voluntarily agreed to participate were included in the study. After an external reviewer confirmed the accuracy of the extracted data, the interviews and coding continued until data saturation was reached. Data saturation was reached after the 10th interview when no new properties or dimensions emerged. Two additional interviews were conducted to confirm that no new data or conceptual codes appeared. The research team and an external reviewer collectively determined saturation through continuous review. In this context, the research sample included 12 ICU nurses.

### Data Collection Tools and Methods

The researchers used an interview form to explore participants’ feelings and experiences. Designed for flexibility and focus, the form contained nine open-ended questions (Table 1).

**Table 1:** Care experience form for COVID-19 ICU nurses – Çanakkale, Türkiye, 2021.

**Questions**
1. Could you explain your working hours in the ICU for COVID-19?
2. Could you describe your nursing care experience for a conscious COVID-19 patient in the ICU?
3. Could you explain your nursing care experience for an unconscious COVID-19 patient in the ICU?
4. Can you describe your emotional experiences related to the nursing care process for patients in the ICU?
5. Could you elaborate on the difficulties you faced during the nursing care process for patients in the ICU?
6. Can you explain your communication process and experiences with patients and their families?
7. Could you describe your experiences regarding communication and teamwork with fellow team members?
8. Can you share your experiences regarding professional ethical principles while caring for COVID-19 patients?
9. Could you talk about the most impactful event during the patient care process in the ICU for COVID-19?

The conceptual framework for the interview questions was defined through a comprehensive review of the literature on nurses’-healthcare experiences during COVID-19^(1,2,3,4,5,6,7,8,11,12,13,14,19,20,21,22,23,24,25,26)^. When creating the interview form, care was taken to ensure the questions were clear and easy to understand, while avoiding leading questions. The aim was to obtain detailed and accurate responses to open-ended questions, both direct and indirect, and to structure the questions in a logical and sequential flow. The interview form began with general questions and easily answerable topics. It was subsequently reviewed by a nursing expert with qualitative research experience for content, scope, and language, and necessary corrections were made. Before initiating the main study interviews, two pilot studies were conducted with individuals from the target population who were not included in the final study group. These pilot studies provided feedback on the clarity and suitability of the questions, as well as suggestions for additional questions. Based on the feedback from these pilot studies, the interview form was revised and finalized before beginning the main interviews. Participants in the study were informed that their personal information and responses would remain confidential and that the data would only be used for research purposes. In-depth interviews were conducted with each participant via the Zoom Cloud Meeting platform, using a semi-structured interview form. Audio and video recordings were made with the participants’ consent. After each question, participants were encouraged to elaborate on their experiences by asking follow-up prompts such as, “What experiences did you have? What happened?” This approach aimed to elicit detailed responses related to the questions. To minimize potential confounding factors, participants conducted the interviews alone at home rather than at work. The interviews continued until participants wished to stop and were designed to feel conversational. On average, the interviews lasted 50 to 75 minutes. During data collection, categorization, and analysis of the data, the real names of the participants were initially used. However, after completing the research, each participant was assigned a number to maintain confidentiality. Following the transcription of the audio recordings into a computer program decoding process, the transcripts were read multiple times to ensure a thorough understanding. During each reading, a conceptual framework was developed to identify potential codes. The interviews were transcribed and coded by the researchers, who analyzed and reported the data in accordance with the Consolidated Criteria for Reporting Qualitative Research (COREQ) (Table 2).

**Table 2:** Consolidated criteria for reporting qualitative research (COREQ): 32-Item – Çanakkale, Türkiye, 2021.

No	Item	Guide questions/Description	Reported on pages #
**Reported on Pages #**			
**Personal Characteristics**			
1	Interviewer/facilitator	Which author/s conducted the interview or focus group?	Methods
2	Credentials	What were the researcher's credentials?	Methods
3	Occupation	What was their occupation at the time of the study?	Methods
4	Gender	Was the researcher male or female?	N/A
5	Experience and training	What experience or training did the researcher have?	Methods
**Relationship with participants**			
6	Relationship established	Was a relationship established prior to study commencement?	Methods
7	Participant knowledge of the interviewer	What did the participants know about the researcher?	Methods
8	Interviewer characteristics	What characteristics were reported about the interviewer/facilitator? e.g. Bias, assumptions, reasons and interests in the research topic	Methods
**Domain 2: Study design**			
**Theoretical framework**			
9	Methodological orientation and Theory	What methodological orientation was stated to underpin the study?	Methods
**Participant selection**			
10	Sampling	How were participants selected? e.g. purposive, convenience, consecutive, snowball	Methods
11	Method of approach	How were participants approached? e.g. face-to-face, telephone, mail, email	Methods
12	Sample size	How many participants were in the study?	Methods
13	Non-participation	How many people refused to participate or dropped out? Reasons?	N/A
**Setting**			
14	Setting of data collection	Where was the data collected? e.g. home, clinic, workplace	Methods
15	Presence of nonparticipants	Was anyone else present besides the participants and researchers?	Methods
16	Description of sample	What are the important characteristics of the sample? e.g. demographic data, date	Methods
**Data collection**			
17	Interview guide	Were questions, prompts, guides provided by the authors? Was it pilot tested?	Methods
18	Repeat interviews	Were repeat interviews carried out? If yes, how many?	N/A
19	Audio/visual recording	Did the research use audio or visual recording to collect the data?	Methods
20	Field notes	Were field notes made during and/or after the interview or focus group?	Methods
21	Duration	What was the duration of the interviews or focus group?	Methods
22	Data saturation	Was data saturation discussed?	Methods
23	Transcripts returned	Were transcripts returned to participants for comment and/or correction?	Methods
**Domain 3: Analysis and findings**			
**Data analysis**			
24	Number of data coders	How many data coders coded the data? The coding process was carried out by both researchers together.	Results
25	Description of the coding tree	Did authors provide a description of the coding tree?	Results
26	Derivation of themes	Were themes identified in advance or derived from the data?	Results
27	Software	What software, if applicable, was used to manage the data?	Methods
28	Participant checking	Did participants provide feedback on the findings?	Conclusion
**Reporting**			
29	Quotations presented	Were participant quotations presented to illustrate the themes / findings? Was each quotation identified? e.g. participant number	Results
30	Data and findings consistent	Was there consistency between the data presented and the findings?	Relationship to existing knowledge
31	Clarity of major themes	Were major themes clearly presented in the findings?	Results
32	Clarity of minor themes	Is there a description of diverse cases or discussion of minor themes?	

Checklist-Çanakkale, Türkiye, 2021.

### Data Analysis

During the interview process, verbal consent was obtained from the participants, and both video and audio recordings were made. The transcription of these recordings was carried out in Microsoft Word files after the interviews. After transcription of each interview, backward translation was conducted by a native Turkish-speaking member of the research team. Each translation was then translated into English again by an English language instructor. The first and second English translations were compared and rechecked. Finally, to ensure translation accuracy, the content was reviewed and approved by an external reviewer fluent in both Turkish and English and experienced in qualitative research. Each interview text was read multiple times, word by word, sentence by sentence, and paragraph by paragraph. Initially, several interviews were conducted, and coding was performed. The extracted codes were then preliminarily classified. Transcription and coding were carried out by the first and second authors. To verify the accuracy of the extracted data, the interviews, coding process, and classified data were reviewed by an external reviewer specializing in qualitative studies. After the external reviewer confirmed the accuracy of the extracted data, the interviews and coding were continued by the research team until data saturation was reached. At the final stage, after achieving data saturation, the extracted codes were reviewed by the research team. Duplicate codes were removed, and categories and subcategories were inductively derived from the initial raw data. To further ensure data accuracy, the external reviewer re-examined the classified data, and the findings were modified based on their feedback. Data analysis was performed using MAXQDA 20. Descriptive analyses, the code theory model, the hierarchical code-subcode model, comparative and relational analyses were employed. In the reporting phase, categories were explained, descriptions were provided, and findings were interpreted.

The research results were supported with direct quotations from participants. To ensure consistency in the results, the coherence of each category and its contribution to a meaningful whole were examined. The collected data were reported in detail, ensuring transparency from data collection to the reporting of findings. Generalizations made at the conclusion of the study were explained as being within the limitations of the results and specific to the studied group. The interview data were presented without interpretation. An independent expert reviewed and confirmed the coding, categories, and analysis. The categorization process began with the in-vivo technique, was then adjusted based on research questions, and finally refined using theoretical concepts. The findings were compared with other studies and supported by relevant literature. In the reporting process, categories were explained, relationships were described, and findings were interpreted. A holistic understanding was achieved by identifying cause-and-effect relationships and drawing conclusions from the findings. During the reporting phase, the researchers re-read the records and selected appropriate quotations to include in the report^(5,6)^.

### Ethical Approvals

Türkiye Ministry of Health (19T14-01-58 approval dated 09/05/2020) and ÇanakkaleOnsekiz Mart University Research Ethics Committee granted ethics approval for this study (2000184619 initial trial approval dated 06/06/2020). Participants gave verbal consent for audio and video recording.

## RESULTS

The participant profiles are presented in Table 3.

**Table 3:** Participants profile – Çanakkale, Türkiye, 2021.

Participants	Age	Sex	Marital status	Education	Who are you living with	Smoking status	Chronic diseases	ICU Experience (Year)	Results of the PCR Test
**P1**	22–30	F	Single	BSc	Family	No	No	1–5	Negative
**P2**	22–30	F	Single	BSc	Alone	No	No	1–5	Negative
**P3**	≥31	F	Married	MSc	Family	No	No	≥ 6	Positive
**P4**	22–30	M	Married	BSc	Family	No	No	< 1	Positive
**P5**	≥31	F	Married	BSc	Family	Yes	No	≥ 6	Negative
**P6**	≥31	F	Married	BSc	Family	No	Yes	1–5	Negative
**P7**	≥31	F	Married	MSc	Family	Yes	No	≥ 6	Negative
**P8**	22–30	F	Single	MSc	Family	No	No	≥ 6	Negative
**P9**	22–30	M	Single	BSc	Alone	Yes	No	1–5	Positive
**P10**	22–30	F	Single	BSc	Alone	No	No	1–5	Positive
**P11**	22–30	F	Single	BSc	Alone	No	Yes	< 1	Negative
**P12**	≥31	F	Married	BSc	Family	No	No	1–5	Negative

A total of 851 primitive codes were obtained. 13 subcodes, 47 codes, 12 categories, and four themes were identified. (a) Categories of the theme “Holistic Care Approach”: barriers, communication, and spiritual care, (b) Categories of the theme “Process Management”: precautions, challenges, adaptation, and treatment processes, (c) Categories of the theme “Physical-Psychological Effects”: physical effects and psychological effects. (d) Categories of the theme “Ethical Dilemma”: ethical behaviors, unethical behaviors, and working principles (Table 4).

**Table 4:** The process of abstracting data – Çanakkale, Türkiye, 2021.

Frame	Themes	Categories	Codes	Sub-codes
**Journey to the ICU During COVID-19 Pandemic**	**Holistic Care Approach**	Barriers	Continuous replacement of PPE Materials supply Fatigue Postponing basic needs The prolonged duration of care	
		Communication	Communication with patients Communication with the patient's family	
		Spiritual Care	Support Morale	
	**Process Management**	Precautions	Individual precautions Institutional precautions	
		Challenges	Ignorance Patient psychology Disease prognosis Uncertainty PPE Risk of transmission/infection Workload Lack of materials Lack of staff Team conflict	
		Adaptation	Team support Team coordination Satisfaction Process contribution Corporate approach	
		Treatment Process	Patient profile Treatment plan Complications Obtained skills Encouragement	
	**Physical-Psychological Effects**	Physical Effects	Health deterioration Headache Exhaustion Sweating	
		Psychological Effects	Negative effects	Sadness Burnout Anxiety/fear Stress Helplessness Feeling bad Hopelessness Disillusionment
			Positive effects	Adaptation Happiness Dedication Spiritual fulfillment Motivation
	**Ethical Dilemma**	Ethical Behaviors	Conscience Equal care Responsibility	
		Unethical Behavior	Patient discrimination Lack of care	
		Working Principles	Integrity Conscience Responsibility Non-discrimination Beneficence	

### (A) Holistic Care Approach

The barriers category has been examined under continuous replacement of PPE, materials supply, fatigue, postponing basic needs and the prolonged duration of care codes. The communication category has been examined under communication with patients and patient’s family codes, and spiritual care category has been examined under support and morale codes.

Many participants reported that the use of PPE prolonged the duration of patient care. They mentioned that the high consumption of PPE and other supplies made the procurement of these materials a time-consuming process. Participants also highlighted that providing care while wearing PPE caused fatigue, excessive sweating, and frequent uniform changes. Some participants noted that they postponed basic needs such as eating and drinking water due to concerns about PPE usage and the fear of infection. Participants also stated that their workload increased due to lack of staff and isolation. Some participants reported that conscious patients remained in constant contact with them due to their inability to communicate with their families. These patients often experienced anxiety and fear of death, which led to frequent interactions with the nurses. Many participants stated that they provided support to patients in various ways.

*“Consumable materials such as PPE are being used in large quantities, and their procurement takes a considerable amount of time.”* (P8)

*“We are always with the patients. Sometimes they want to hold our hand. The patient psychologically wants you to be there. They say, ‘Don’t go.’”* (P9)

*“The area gets very hot. You are forced to sweat. How many times do you change clothes? You get thirsty, but you can’t drink water, and when you take a break to drink, you need to go to the restroom during care.”* (P11)

*“The care of the patient varies according to the patient’s condition. If the patient is intubated, the care takes a long time. When we take a break, we constantly change the PPE and uniforms.”* (P12)

When the theme of the Holistic Care Approach was evaluated based on the participants’, it was determined that their views predominantly focused on the support. Furthermore, when this theme was evaluated according to participants marital status’, single nurses expressed more intense views regarding communication with patients compared to married nurses.

### (B) Process Management

The precautions category has been examined under individual precautions and institutional precautions codes. Challenges category has been examined under ignorance, patient psychology, disease prognosis, uncertainty, PPE, risk of transmission/infection, workload, lack of materials, lack of staff and team conflict codes. The adaptation category has been examined under team support, team coordination, satisfaction, process contribution and corporate approach codes, and the treatment process category has been examined under patient profile, treatment plan, complications, obtained skills and encouragement codes.

In the precautions category, participants shared their experiences regarding the use of PPE and the fatigue caused by wearing it. In the challenges category, the most frequently reported issue identified by participants was workload. The participants attributed the increase in their workload to a lack of staff. They also stated that they were often required to perform tasks belonging to other disciplines for various reasons, further increasing their workload. Many participants reported difficulties collaborating with both nurses and other team members.

*“I couldn’t change my patient’s position every two hours. Why? Because there is a lack of staff. I can’t do this alone.”* (P2)

*“Our care processes usually take a long time. We call it the black hole. We stay here for at least 3 to 4 hours.”* (P3)

*“What I struggle with the most is related to vision. Because I have my own glasses, unfortunately. When I put on protective glasses and a face shield, after half an hour, they fog up. Since I can’t leave the area, I have to continue like that.”* (P6)

*“Even though we had patients we were taking care of, including oral care, we had many conflicts when physicians didn’t come into the ICU, observed from afar, or gave verbal orders over the phone.”* (P7)

In the adaptation category, the most frequently mentioned code was process contribution. Participants mentioned that working in compliance with the team sometimes helped reduce the workload. Additionally, some participants expressed positive feelings and satisfaction during the pandemic. They reflected on how working in the COVID-19 ICU enriched their professional experience and allowed them to witness a historical process.

*“We try to take turns on patient care. But sometimes, we all have to get involved in care. We have care three times a day, and we dedicate approximately 16 hours of the 24-hour shift to patients.”* (P7)

*“The patient said, ‘I will never forget you. If you hadn’t provided me with this psychological support, I don’t know if I could have been discharged from here like this.’”* (P8)

In the treatment process category, participants indicated that many patients were young and did not have chronic illnesses. Participants also reported that pressure injuries often occurred due to patient positioning and the equipment used. Some patients struggled to tolerate the prone position. Additionally, some participants stated that they gained valuable experience during the work and applied it to the treatment of other patients.

*“We tried to prevent pressure injuries by constantly changing the position of patients lying face down in the chest, chin, and forehead areas.”* (P1)

*“Patients’ general condition can deteriorate very quickly. A conscious patient can suddenly arrest. Conscious patients are also afraid. You talk to them. You become their hope.”* (P12)

When the theme of process management was evaluated based on the participants’, it was determined that their views were primarily focused on the duration of care. Furthermore, when this theme was evaluated according to participants ICU working experience, participants with 1–5 years of ICU working experience expressed more intense views regarding PPE.

### (C) Physical-Psychological Effects

The physical effects category was examined under the codes of health deterioration, headache, exhaustion, and sweating.

Participants stated that the PPE they used caused headaches and excessive sweating. Some participants reported experiencing illness and hair loss, while others expressed feeling physically exhausted during their work.

*“My hair used to fall out very little before. Now, it has started falling out excessively due to overwork. I’ve also started waking up screaming at night. Additionally, positioning patients physically exhausted me.”* (P4)

*“Wearing layered masks caused me severe headaches.”* (P5)

*“I sweat constantly due to using PPE, but I also have to take care of the patient.”* (P12)

The psychological effects category has been examined under negative and positive effects. The negative effects code has been examined under sadness, burnout, anxiety/fear, stress, helplessness, feeling bad, hopelessness and disillusionment sub- codes Table 4. In the negative effects code, the most intensely expressed emotion was sadness. Participants described the emotional toll of frequent patient losses, particularly of younger patients, as well as the burden of excessive workloads. Many participants reported feeling distressed due to the unfair distribution of tasks and their heavy workload. They also shared feelings of burnout and fear, particularly regarding the risk of contracting COVID-19 or transmitting it to their families. Additionally, some participants expressed frustration with insufficient PPE, which made them feel inadequately protected, and feelings of hopelessness when patients failed to recover.

The positive effects code has been examined under adaptation, happiness, dedication, spiritual fulfillment and motivation sub-codes. However, participants also mentioned moments of happiness and motivation, particularly when patients recovered, when they witnessed the dedication of their colleagues, and when they experienced spiritual fulfillment through their work. Many participants expressed pride in their commitment to their profession during the pandemic.

*“Right now, it’s the worst period I’ve ever experienced in my profession... We are helpless. We are scared. We are sad... The difficulties we face in care due to staff shortages are significant. Motivation decreases. You become unhappy.”* (P3)

*“Nursing gives me great pleasure. When a patient says ‘thank you,’ it motivates me incredibly.”* (P6)

*“In the ICU, all of the patient’s care, their psychological support, physical needs, and overall burden fall on the nurses. The patient has no one else to rely on. They are completely dependent on you, like a baby. We also experienced fulfillment through this responsibility.”* (P11)

When the theme of physical-psychological effects was evaluated based on the participants’, sadness and burnout emerged as the most commonly expressed views. Furthermore, when this theme was evaluated according to participants’ COVID-19 test results, participants with negative COVID-19 test results experienced more intense negative emotions compared to those who had tested positive.

### (D) Ethical Dilemma

The ethical behaviors category has been exmined under conscience, equal care and responsibility codes, the unethical behavior category has been exmined under patient discrimination and lack of care codes, and the working principles category has been exmined under integrity, conscience, responsibility, non-discrimination and beneficence codes.

In this theme, participants expressed strong opinions in the conscience code, emphasizing their efforts to provide equal care to all patients without discrimination. Additionally, they discussed their moral and emotional experiences during the patient care process. In the unethical behaviors category, participants mentioned several factors contributing to inadequate care, including a lack of staff, insufficient PPE, physicians not entering the ICU, and some nurses refusing to provide care due to the risk of infection. In the working principles category, the most prominent code identified was responsibility. Many participants stated that they consistently upheld the principle of providing equal and fair care to all patients, regardless of the circumstances.

*“I pay attention to providing equal and fair care to all patients.”* (P10)

*“I cannot say with a clear conscience that I have completed all care for my patients. I couldn’t provide the necessary positioning for my patient when needed. I couldn’t provide sufficient care to prevent pressure injuries or for developing patients.”* (P3)

*“Patients were deprived of adequate care. We couldn’t provide sufficient care to the patients.”* (P5)

*“Patients didn’t choose to have this illness knowingly. I shouldn’t provide inadequate care to them. Regardless of the patient’s race, gender, or age, I should provide care.”* (P10)

When the theme of Ethical Dilemma was evaluated based on the participants’, participants predominantly expressed their concerns about the lack of adequate care. Furthermore, when this theme was evaluated according to participants’ age, participants under 30 years old expressed more intense views about conscience and equal care, while participants aged 31 years or older expressed stronger concerns about inadequate care.

**Figure 1:**
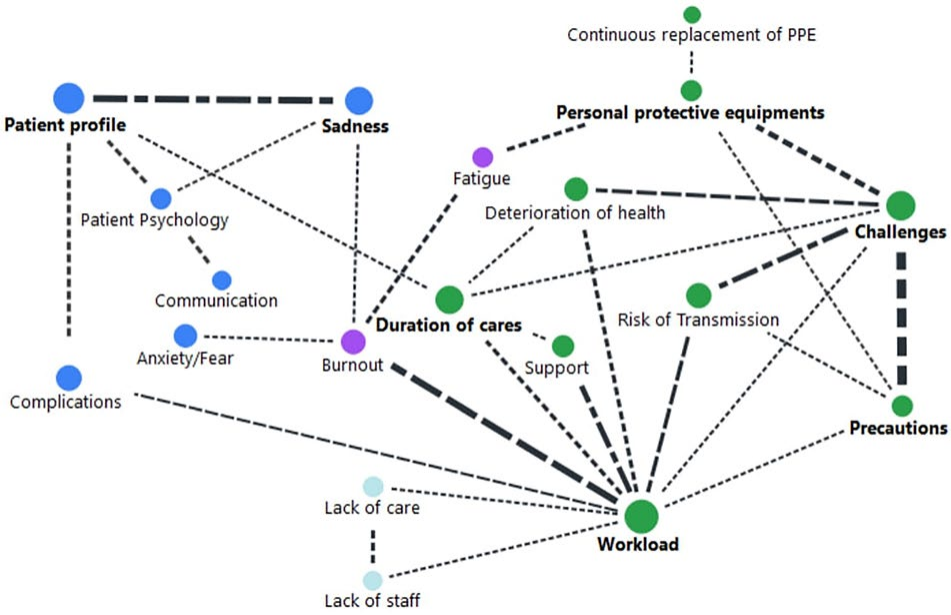
Code map-Çanakkale, Türkiye, 2021.

Relational analyses emerged from the convergence of participants’ opinions. Participants not only shared their challenges but also discussed other related issues, such as PPE shortages, precautions, workload, care durations, and health deterioration (Figure 1).

A participant summarized the situation as follows:

*“For the care and treatment process of each patient, we wear our gown, cap, N95 mask and surgical mask, visor, overshoes... The care process takes at least 1.5 hours for an intubated patient. Oral care, endotracheal tube aspiration, tube ligature change, giving the prone position, pressure injury assessment, and so on. For some patients, care takes three hours. Position changes are every two hours. We also provide psychological support and moralization for conscious patients. It’s all very difficult; our physical and mental health has deteriorated.”* (P4)

## DISCUSSION

Study data were collected between April- September 2021, a period corresponding to the post-vaccination yet still high- risk phase of the COVID-19 pandemic in Türkiye. During this time, ICUs remained under substantial pressure due to fluctuating infection rates and the ongoing treatment of critically ill patients. Although the initial phase of acute uncertainty had subsided, concerns about viral variants, PPE shortages, and prolonged workloads persisted. The timing of data collection therefore reflects a phase characterized by sustained clinical burden, increasing professional fatigue, and gradual adaptation to long-term crisis conditions. In the literature, healthcare professionals faced numerous challenges, including pathogen exposure, increased workloads, and extended working hours, all of which contributed to fatigue, professional burnout, and psychological distress^(1,2)^. Caring for COVID-19 patients was particularly demanding for nurses, with documented impacts such as decreased appetite, sleep disturbances and fatigue. Furthermore, the use of PPE was reported to cause physical discomfort, restrict movement, create feelings of suffocation, induce excessive sweating, delay the basic needs, and result in dehydration and constipation^(3,4,5,6,7)^.

In our study, participants similarly described working with PPE as physically and mentally challenging. They reported prolonged nursing care sessions without adequate rest, leading to fatigue. Additional concerns included excessive sweating, frequent uniform changes, and the postponement of basic physical needs. Participants also noted that workloads increased due to staff shortages and the reluctance of some personnel to enter ICU areas out of fear of infection. The literature indicates that factors such as reduced staffing in ICU, attempts to deliver the same level of care with fewer personnel, and the demands of using PPE and adhering to isolation measures negatively impact holistic patient care^(3)^.

In our study, participants emphasized the importance of a holistic and comprehensive approach to patient care, prioritizing psychological needs alongside physical treatment. Participants reported that patient isolation in the ICU, lack of family contact, and the severity of illness heightened patients’ anxiety. To mitigate this, participants described providing emotional support by holding patients’ hands, speaking to them, and offering moral encouragement to reduce their fear of death and alleviate anxiety. As the only connection between patients and their families, nurses played a critical role in addressing patients’ emotional needs. Consistent with these findings, the literature highlights that spiritual support provided by healthcare staff is crucial for COVID-19 patients, with nurses often placing themselves at risk to strengthen nurse- patient relationships despite the stressful environment^(2,15)^.

Another study reported that nurses, as the first point of contact with patients, often extended warmth and care despite their own fatigue and anxiety, treating patients as though they were family members^(5)^. Similarly, in our study, participants frequently communicated with conscious patients, helping to alleviate their fears and incorporating patient education into their care processes. However, due to limitations in ICU technological infrastructure, participants could not facilitate direct communication between patients and their families. Instead, they took responsibility for informing families about patients’ conditions.

In our study, participants frequently highlighted the lack of PPE as a significant challenge during the care process. They noted that the absence of adequate PPE and personnel increased both the duration of patient care and their overall workload. Participants also reported that some team members avoided performing their duties by not entering the ICU due to fear of infection. Additionally, participants expressed concerns about contracting the virus themselves and potentially infecting their loved ones. The uncertainty surrounding the duration of the pandemic and the poor prognosis of many patients further exacerbated their stress and emotional burden. In the literature nurses have reported issues such as a lack of PPE, inadequate protective measures, and insufficient PPE usage guidelines, alongside increased workloads due to staff shortages. Although COVID-19 patients present with a wide range of clinical conditions, studies have documented that even young patients without pre-existing comorbidities can experience sudden clinical deterioration, often necessitating mechanical ventilator support^(3,6,27)^. In our study participants observed that patients without chronic illnesses could experience rapid declines in their conditions. The frequent mention of genetic patient profiles and mortality by participants suggests the potential development of compassion fatigue in this context. Despite these challenges, some participants noted that working under such demanding conditions provided them with unique professional experiences and fostered teamwork. Consistently, the literature indicates that nurses reported improvements in their isolation practices, communication skills, and overall professional growth during the pandemic^(28)^

In our study participants reported experiencing significant physical and psychological challenges, including headaches, fatigue, stress, and anxiety. These challenges arose from prolonged PPE use, workloads, and the risk of disease transmission during the care process. Consistent with these findings, previous research has shown that nurses experienced notably high levels of occupational fatigue during the pandemic^(14)^. The use of PPE has been associated with various physical discomforts, including headaches, respiratory difficulties, panic attacks, dermatitis, and allergic reactions^(4,7)^. A study reported that wearing PPE restricted mobility, elevated body temperature, increased sweating, and created a sensation of suffocation^(5)^. In other study participants reported decreased appetite, fatigue, and difficulty in sleeping as common experiences^(8)^. Physical fatigue was linked to the isolation process and PPE use, with continuous wear causing excessive sweating, dehydration, and exhaustion during extended care periods^(9)^.

Psychological effects have also been widely documented in the literature. Nurses frequently reported fear of contracting the virus, hesitance to interact with family members, and feelings of insecurity. These experiences were compounded by stress, anxiety, depression, confusion, irritability, and restlessness. The inability to prevent rapid patient deterioration and frequent patient losses led to compassion fatigue, along with feelings of sadness, anger, guilt, pessimism, hopelessness, and emotional exhaustion. Nurses also described losing motivation and experiencing a sense of meaninglessness in their work^(6,8)^. In a study, participants expressed intense anxiety while caring for patients in isolation areas, as well as fear of becoming infected and infecting their families. Concerns about managing large numbers of patients with insufficient staff were also commonly reported^(15)^. Witnessing patient suffering, feeling helpless to alleviate it, and observing the deaths of young patients were particularly distressing for nurses, with some describing these experiences as unbearable^(5)^.

Our findings corroborate these observations. In this study participants experienced both severe physical and psychological effects throughout the caregiving process. Despite these challenges, participants described the recovery of patients as a source of immense joy, often likening it to a miracle. They noted that the gratitude expressed by recovering patients served as a strong motivational factor, inspiring them to strive for the best possible care. Similarly, the literature highlights that nurses, despite their fatigue and personal struggles, often treat their patients with the same care and attention they would give a family member, without expecting anything in return^(5)^.

Our study revealed that most participants reported being unable to provide adequate care due to factors such as staff shortages and lack of materials. Similarly, a study found that reductions in staffing increased nurses’ workloads, while newly assigned nurses struggled to deliver quality care due to insufficient knowledge^(3)^. At present study participants also described feeling abandoned in the ICU, stating that the care they provided to patients was limited to what they could manage, which caused them significant moral distress. A study reported that although nurses spent extended periods with patients during the care and treatment process, physicians were often absent, equality principles were not adhered to, and nurses frequently had to perform procedures typically carried out by physicians^(11)^. Similarly results found that the quality of care in COVID-19 ICUs declined due to increased workloads and reduced personnel. The literature further indicates that the combination of transmission risk, cessation of treatment for critically deteriorating patients with high mortality risk, and shortages of staff and PPE often forces nurses to provide incomplete care^(12,13)^. This includes compromising patient safety and quality of care, as well as neglecting routine nursing activities such as oral care, prevention of pressure injuries, mobilization, and delirium screening in the ICU^(13)^.

In our study, some participants tried to stay with patients longer to meet all their needs, while others felt guilty about not being able to provide adequate care. Participants also reported having to choose between the patients’ needs and their own safety on multiple occasions. Nonetheless, participants emphasized that, to them, all patients are equal and that they strive to ensure that treatment, care, and facilities are as equitable as possible. The literature highlights that nurses often feel unable to provide adequate care and, at times, face Ethical Dilemma where they must choose between prioritizing their own well-being or that of their patients. Interestingly, many nurses reportedly fail to recognize these dilemmas as ethical issues^(5,29)^. In our study, participants reported applying the principles of beneficence, non-maleficence, and justice in their care processes. However, despite the foundational goal of nursing being to alleviate suffering and provide quality care, several participants acknowledged that they could not deliver adequate care due to various challenges encountered during the care process. Some participants also reported that working in the COVID-19 ICU was professionally beneficial, providing them with unique learning opportunities and new perspectives. Similarly, the literature suggests that the pandemic offered unexpected professional growth for many nurses, including a deeper understanding of their profession, increased resilience, experience in managing COVID-19 patients, and enhanced crisis management skills^(28,30)^.

The temporal context of data collection also influenced ICU nurses’ reported experiences and perceptions of risk. Conducting interviews during the mid-to-late phase of the pandemic meant that participants had already developed adaptive coping mechanisms and greater familiarity with COVID-19 treatment protocols. However, they also reported cumulative fatigue and emotional exhaustion from prolonged exposure to crisis conditions. Unlike studies conducted during the initial outbreak phase, where fear of infection and uncertainty dominated^(2,6,15)^, the participants in this study described a complex blend of chronic strain, moral distress, and professional endurance. This contextual nuance is essential for interpreting the findings, as it situates Turkish ICU nurses’ experiences within a dynamic trajectory of adaptation, resilience, and continuing workload stress.

The findings of this study underscore the need for institutional policies that prioritize the psychological well-being, physical safety, and professional sustainability of ICU nurses, particularly in crisis conditions such as pandemics. Institutions should adopt a proactive and multidimensional approach that integrates continuous psychological support, structured debriefings, and flexible staffing policies to foster resilience and enhance the quality of care. Given the documented emotional strain, sadness, and burnout experienced by ICU nurses, hospitals should implement permanent, accessible psychological support systems. These could include regular mental health check-in, counseling services, and peer-support groups led by trained psychologists or mental health nurses. Integrating mental health promotion into daily clinical practice through stress management workshops, resilience training, and mindfulness-based interventions can reduce long-term psychological sequelae and increase professional satisfaction.

Structured debriefing sessions following critical incidents, patient losses, or extended high-intensity shifts allow nurses to express their experiences, process moral distress, and derive shared meaning from their work. Such sessions not only mitigate emotional exhaustion but also contribute to a culture of psychological safety and team cohesion. Regular reflective practice meetings can also help identify systemic challenges, foster mutual learning, and inform managerial decision- making. The results highlight how staff shortages, prolonged care durations, and excessive workloads compromised both nurse and patient safety. Institutions should develop flexible staffing models that account for surges in patient volume and enable equitable workload distribution. Policies promoting rest breaks, rotational scheduling, and backup staffing during pandemics or other crises can alleviate fatigue and improve retention. Training programs can also enhance staff adaptability across units, reducing the strain on ICU nurses. Effective leadership communication and visibility are critical in times of crisis. Nursing managers should adopt participatory management approaches that validate nurses’ experiences, encourage feedback, and involve staff in policy formation. Institutions should also ensure transparent communication about PPE availability, safety protocols, and evolving guidelines to reduce uncertainty and moral distress.

Hospitals must treat ICU nurses’ well-being as an organizational priority equivalent to patient safety. Establishing wellness committees, recognition programs for frontline workers, and reward systems for teamwork and ethical practice can foster morale and reinforce professional identity. Furthermore, integrating mental health metrics and staff feedback into institutional quality improvement frameworks can ensure sustainability and accountability. By embedding these measures into institutional structures, healthcare organizations can not only mitigate the psychological and ethical burdens reported in this study but also strengthen workforce resilience, retention, and quality of care during future public health emergencies.

The experiences of Turkish ICU nurses during the COVID-19 pandemic align with global findings but also reflect unique contextual features rooted in Türkiye’s healthcare infrastructure and cultural values. Similar to studies from Spain, Iran, and Italy, Turkish nurses reported excessive workloads, PPE shortages, emotional exhaustion, and moral distress linked to staff shortages and rapidly evolving treatment protocols^(3,6,17,18,22)^. Across all these contexts, ICU nurses demonstrated strong professional commitment and adaptability despite inadequate systemic support. However, the Turkish reality also presents distinct dimensions that merit attention. The rapid expansion of ICU capacity during the pandemic in Türkiye created increased staffing demands that were not always matched by human resource support. This led to heightened workloads, prolonged care durations, and more frequent ethical tensions. Unlike in some countries, where structured psychological support and institutional debriefing systems were established early in the pandemic^(3,21)^, Turkish ICU nurses often relied on informal peer solidarity and personal coping mechanisms. Moreover, the collectivist cultural context, characterized by a strong sense of duty and moral responsibility, appeared to reinforce nurses’ ethical commitment and their tendency to continue care despite personal risk. This sense of moral obligation parallels findings from studies in other contexts^(6,19)^, where social and professional responsibility norms similarly shaped nurses’ responses to crisis care. By situating the Turkish findings within the global landscape, the study demonstrates both the universality and the contextual specificity of ICU nurses’ experiences during COVID-19. The shared challenges across diverse health systems highlight the need for international collaboration in developing evidence-based institutional policies particularly in strengthening psychological support systems, ensuring adequate staffing, and protecting nurses’ ethical and physical integrity during health emergencies. The Turkish case underscores that even in middle-income countries with rapidly mobilized healthcare responses, structural limitations can hinder the sustainability of holistic care. Thus, lessons learned from Türkiye may inform both regional and global strategies for enhancing critical care resilience.

Since the participants were predominantly employed in the western and northwestern provinces of Türkiye, the generalizability of the findings is limited. In addition to the limited geographical representation of participants, most of whom were employed in western and northwestern Türkiye. This study faced methodological constraints associated with the use of online interviews. Conducting data collection via the Zoom Cloud Meeting platform, while necessary during pandemic restrictions, may have influenced the depth and spontaneity of participant responses. The virtual format might have reduced the sense of interpersonal connection, limited opportunities for rapport-building, and constrained the researchers’ ability to observe non-verbal cues such as facial expressions, posture, and emotional nuances, which are valuable in phenomenological inquiry. Although verbal descriptions were rich and reflective, the absence of full non-verbal observation may have affected the interpretation of emotional intensity or subtle communication patterns. Furthermore, the reliance on self-reported experiences introduces the possibility of recall bias or social desirability bias, as participants may have moderated their responses to align with professional norms.

### Implications for Clinical Practice and Policy Development

The study emphasizes that physical, psychological, and environmental factors collectively undermine the quality of care and the holistic nursing process, posing risks to both nurse and patient safety. It underscores that providing adequate institutional support including sufficient staffing, mental health services, and protective organizational policies is essential for maintaining safe and holistic care delivery in intensive care settings. The findings also reveal that ICU nurses experience significant moral and ethical distress due to systemic barriers that limit their ability to deliver complete and equitable care. Particularly during health crises such as pandemics, challenges such as fatigue from prolonged PPE use, staffing shortages, and postponed basic needs highlight the necessity for institutional reforms and revised clinical protocols to better support nurses and safeguard patient outcomes.

#### 1. Revising Protocols to Minimize Fatigue from PPE Use

Given that prolonged use of PPE caused headaches, excessive sweating, dehydration, and delays in meeting basic needs, hospitals should revise existing infection control protocols to balance safety with physiological needs. Structured rotation systems can be introduced, whereby staff alternate between high-exposure and low-exposure zones, allowing scheduled intervals for rest, hydration, and changing PPE. Establishing designated “recovery zones” near ICU units where nurses can safely remove PPE, rehydrate, and cool down before returning to care can substantially reduce fatigue and improve concentration. Furthermore, the introduction of improved PPE materials lightweight, breathable, and ergonomically designed should be prioritized in procurement policies to enhance comfort without compromising safety.

#### 2. Adjusting Staffing Schedules to Allow Regular Breaks and Hydration

The study findings demonstrate that staff shortages and prolonged care durations often led to physical exhaustion and decreased motivation. Institutions can address this by adopting more flexible and responsive staffing models. Scheduling systems should ensure protected time for rest and nutrition, even during peak patient loads. Managers can use workload-tracking systems or “acuity-based staffing” models to distribute assignments fairly and in real time, preventing burnout and ensuring equitable care delivery. Additionally, implementing short but frequent microbreaks within shifts such as 20 minutes every 4 hours can significantly improve focus, reduce fatigue, and maintain patient safety.

#### 3. Embedding well-being and Mental Health Support into Daily Practice

Beyond physical fatigue, nurses in this study reported sadness, helplessness, and emotional exhaustion. To address this, hospitals should integrate psychological support mechanisms into everyday operations, rather than treating them as emergency interventions. Regular on-site counseling sessions, peer- support circles, and post-shift debriefings can provide emotional relief and foster resilience. Embedding trained mental health professionals within ICU teams can also help identify early signs of distress and provide timely support.

#### 4. Enhancing Interdisciplinary Teamwork and Leadership Responsiveness

Participants’ experiences highlighted that team conflicts and inconsistent managerial support intensified workload stress. Encouraging transparent communication, participatory decision-making, and mutual accountability among interdisciplinary team members can strengthen morale. Nursing leaders should conduct regular briefings to review care challenges, gather feedback, and communicate changes in PPE, staffing, or workflow policies clearly.

#### 5. Integrating Feedback into Continuous Quality Improvement

Hospitals can institutionalize mechanisms for nurses to report fatigue-related concerns, PPE discomfort, or ethical challenges anonymously. Feedback collected from frontline staff can inform continuous improvement cycles, leading to more responsive protocols and greater organizational trust. By translating these insights into operational policies, healthcare institutions can move beyond reactive crisis management toward a more sustainable model of holistic, human-centered care. Reducing physical strain, ensuring adequate rest, and embedding psychological support are not only measures to protect nurses but also critical steps in safeguarding patient safety and preserving the integrity of intensive care practice.

## CONCLUSION

The concept of “The journey to the ICU during the COVID-19 pandemic,” as framed in this study, captures the transition of ICU nurses from negative and distressing experiences to moments of happiness and professional fulfillment. The findings reveal that COVID-19 ICU nurses encountered a wide spectrum of care experiences, encompassing both negative and positive aspects. Negative care experiences pose risks to both nurse and patient safety. Positive experiences serve as a source of motivation and hope, encouraging nurses to continue providing care. Factors such as increased workload, fatigue, staff shortages, lack of equipment, risk of infection, physical and psychological inconvenience from PPE use, insufficient team support, and the inability to provide comprehensive care can compromise safety and the overall quality of care. Addressing these issues is critical to safeguarding the well-being of nurses and patients alike. Providing adequate support for ICU nurses can empower them to effectively develop and implement holistic care models.

The study highlighted both weaknesses and strengths within the healthcare system based on the care experiences. Nurses play a critical role in caring for critically ill patients. Nursing care has been significantly affected by factors such as fear, isolation, staff shortages, inadequate PPE, and etc., which have posed challenges to maintaining the humanization of healthcare. The findings suggest that fostering nurse and patient safety during the care process and addressing challenges encountered in the work environment can positively influence nursing practices. Enhancing the safety of ICU nurses not only improves patient safety but also contributes to the delivery of holistic care at an optimal level. Additionally, it is anticipated that such measures will improve nurse satisfaction and strengthen their commitment to the profession.

## Data Availability

The entire dataset supporting the results of this study is available upon request to the corresponding author.
