# Supplementary material for: Exploration of Turkish ICU Nurses’ care experiences during the COVID-19 pandemic: A qualitative study
Source: Rev Esc Enferm USP. 2026 Jan 30;60:e20250274. doi: 10.1590/1980-220X-REEUSP-2025-0274en (PMC12857919; doi:10.1590/1980-220X-REEUSP-2025-0274en)
Supplement: Translation

# Translation: Exploración de las experiencias asistenciales de las enfermeras de cuidados intensivos turcas durante la pandemia de COVID-19: un estudio cualitativo

**DOI:** 10.1590/1980-220X-REEUSP-2025-0274es

**Authors:** Demet Acar, Araz Askeroğlu

**Affiliations:** 1 Çanakkale Onsekiz Mart University Hospital, Department of General Surgery, Çanakkale, Turkey.; 2 Çanakkale Onsekiz Mart University, Faculty of Health Sciences, Department of Surgical Nursing, Çanakkale, Turkey.

**Keywords:** COVID-19, Unidades de Cuidados Intensivos, Enfermería, Seguridad del Paciente

## Abstract

**Objetivo:**

estudio fenomenológico tuvo como objetivo describir las experiencias de cuidado de las enfermeras turcas de UCI durante la pandemia de COVID-19.

**Método:**

Los datos se recopilaron mediante entrevistas semiestructuradas con 12 enfermeras de UCI. Este estudio se llevó a cabo en hospitales públicos, que fueron los principales centros de la pandemia en Turquía. Los datos fueron analizados mediante métodos descriptivos, comparativos y de análisis relacional.

**Resultados:**

Se identificaron un total de 851 códigos primarios. Se determinaron cuatro temas, 12 categorías, 47 códigos y 13 subcódigos: (a) Categorías del tema “Enfoque de cuidado holístico”: barreras, comunicación y cuidado espiritual. (b) Categorías del tema “Gestión del proceso”: precauciones, desafíos, adaptación y procesos de tratamiento. (c) Categorías del tema “Efectos físicos-psicológicos”: efectos físicos y efectos psicológicos. (d) Categorías del tema “Dilema ético”: conductas éticas, conductas no éticas y principios de trabajo. En el análisis relacional, las participantes expresaron sus opiniones respecto de desafíos, así como precauciones, carga laboral, duración del cuidado y deterioro de la salud.

**Conclusión:**

Las participantes informaron experiencias de cuidado tanto positivas como negativas. Las experiencias negativas pueden afectar de forma adversa la seguridad del paciente, mientras que las experiencias positivas actúan como fuente de esperanza y motivación, incentivando a las enfermeras a continuar proporcionando un cuidado holístico.

## INTRODUCCIÓN

En marzo de 2020, la Organización Mundial de la Salud (OMS) destacó que los profesionales sanitarios que participaban en la lucha contra la COVID-19 se enfrentaban a numerosos riesgos, entre ellos la exposición a patógenos, cargas de trabajo excesivas, fatiga, agotamiento profesional y violencia física y psicológica. La repentina y rápida aumento de la demanda de atención médica causada por la pandemia de COVID-19 supuso una carga significativa para los sistemas de salud de todo el mundo^(1,2)^. La literatura indica que las enfermeras que atienden a pacientes diagnosticados con COVID-19, a menudo se enfrentan a retos importantes, como la sensación de agobio y agotamiento debido a la carga de trabajo y la obligación de llevar equipo de protección personal (EPP) de forma continua. Además, las enfermeras expresaron su preocupación por la insuficiencia de los EPP, el riesgo de contraer el virus y la posibilidad de transmitir la enfermedad a sus familias. A estos problemas se suman las preocupaciones sobre su capacidad para proporcionar la atención adecuada y necesaria a los pacientes, junto con trastornos físicos y psicológicos como sudoración excesiva, sensación de fatiga, ansiedad e impotencia^(1–6)^. Los estudios han demostrado que, aunque el uso de EPP era obligatorio para quienes trabajaban en las UCI de COVID-19, causaba estrés, ansiedad y diversos problemas físicos y psicológicos^(7,8)^. Además, las enfermeras de las unidades de cuidados intensivos (UCI) se enfrentaron a importantes retos mientras trabajaban en las unidades de COVID-19, entre ellos la necesidad de posponer sus necesidades básicas y sus cargas de trabajo, así como otras dificultades^(3,5,9,10)^. Aunque las enfermeras de las UCI de COVID-19 son el primer punto de contacto con los pacientes, se han identificado factores como la carga de trabajo, la escasez de personal, el riesgo de infección y el uso de EPP como retos que impiden a las enfermeras proporcionar una atención integral a los pacientes^(5,11–13)^. Además, las enfermeras, que son el único vínculo entre los pacientes y sus familias, desempeñan un papel fundamental a la hora de atender las necesidades emocionales de los pacientes^(14)^. La literatura destaca que el apoyo espiritual que brindan las enfermeras es crucial para los pacientes con COVID-19^(2,15)^.

La COVID-19 sometió a los trabajadores sanitarios, en particular a las enfermeras, a un estrés intenso en Turquía, al igual que en todo el mundo. El número de camas de cuidados intensivos en Turquía aumentó durante la pandemia de COVID-19, lo que llevó a las enfermeras de las UCI a trabajar en condiciones extremadamente duras. Se expresó preocupación por el mayor riesgo de contraer COVID-19 al que se enfrentaban las enfermeras, ya que el aumento del número de pacientes y la falta de personal contribuían a la sobrecarga de trabajo, la fatiga, los conflictos de comunicación entre el equipo y la disminución de la calidad de la atención prestada^(16)^.

Los estudios que examinan las experiencias de las enfermeras de la UCI durante la pandemia de COVID-19 destacan importantes retos físicos, psicológicos y profesionales. Un estudio reveló que las enfermeras de la UCI se enfrentaban a un estrés debido a la falta de tratamientos basados en la evidencia, los malos pronósticos de los pacientes, el apoyo inadecuado de los directivos y el respaldo insuficiente de la comunidad, lo que suponía una carga inmensa, entre otras cosas. El estudio hizo hincapié en la necesidad de seguir investigando para comprender mejor las experiencias de las enfermeras y desarrollar recursos que apoyen su bienestar^(17)^. Un estudio de revisión que evaluó el impacto de la pandemia en el personal de enfermería de las UCI identificó las áreas clave afectadas, entre ellas el aumento de la carga de trabajo, los cambios en la organización del personal de enfermería, los retos a los que se enfrentaba el personal reasignado, las percepciones sobre la seguridad de los pacientes y la calidad de la atención, y los efectos sobre la salud del personal de enfermería^(18)^. Del mismo modo, un estudio cualitativo reveló que el personal de enfermería de las UCI experimentó intensos efectos psicológicos y físicos, al tiempo que se enfrentaba a los cambios sociales y a las diferentes percepciones del público sobre la pandemia^(19)^. En Irán, una investigación que examinó los retos a los que se enfrentaban las enfermeras de la UCI al atender a pacientes con COVID-19 identificó el estrés psicológico, el agotamiento físico, las ineficiencias organizativas y la incertidumbre sobre la enfermedad como las principales preocupaciones. El estudio destacó la importancia de comprender estos retos para que las autoridades sanitarias puedan aplicar las intervenciones adecuadas, mejorar las instalaciones sanitarias, apoyar al personal y proporcionar información basada en pruebas y apoyo psicológico^(6)^. Un estudio cualitativo realizado en España exploró las experiencias y percepciones del personal de enfermería de las UCI durante la pandemia, identificando tanto los puntos fuertes como los débiles del sistema sanitario. El estudio reveló que el miedo y el aislamiento influyeron en la atención de enfermería, lo que dificultó el mantenimiento de prácticas sanitarias humanizadas. También subrayó la necesidad de optimizar la gestión de los recursos, el apoyo psicológico, la formación adecuada y el desarrollo de protocolos de alta calidad para futuras emergencias^(3)^. Una revisión de alcance que investigó el agotamiento entre el personal de enfermería de las UCI identificó altos niveles de agotamiento, con factores contribuyentes como la falta de equipo, el estigma social, el aumento de la carga de trabajo y el miedo al contagio. Sin embargo, se descubrió que el apoyo social y el reconocimiento profesional eran factores protectores significativos contra el agotamiento^(20)^. Otro estudio realizado en España informó de que el personal de enfermería de cuidados intensivos y de urgencias se enfrentó a cargas de trabajo excesivas, altas proporciones de pacientes por enfermero y agotamiento emocional durante toda la pandemia^(9)^. Otras investigaciones indicaron que las enfermeras de la UCI se enfrentaban a retos a la hora de colaborar con nuevos compañeros, mantener las relaciones existentes en el lugar de trabajo y la falta de reconocimiento institucional, lo que contribuía al agotamiento emocional y físico^(21)^. En Irán, entre los retos adicionales señalados por las enfermeras de la UCI figuraban la desconexión de la enfermería holística, las ineficiencias organizativas, el agotamiento laboral y la evolución de la dinámica del lugar de trabajo^(22)^. A pesar de estos retos, algunos estudios destacaron la resiliencia, la adaptabilidad y el fuerte liderazgo de las enfermeras de UCI en la gestión de la atención a los pacientes durante la crisis^(23,24)^. Sin embargo, la seguridad de los pacientes y la calidad de la atención se vieron a menudo comprometidas, lo que provocó estrés ético y efectos adversos en el bienestar físico y psicosocial de las enfermeras^(25)^. Los resultados de un estudio hicieron hincapié en el papel fundamental del trabajo en equipo y el apoyo de los compañeros para ayudar a las enfermeras de UCI a hacer frente a las condiciones extremas durante la pandemia^(26)^. Según los resultados de la revisión bibliográfica, la pandemia de COVID-19 supuso una presión considerable para el personal de enfermería de las UCI, afectando a su carga de trabajo, su bienestar psicológico y la calidad de la atención al paciente. El objetivo del estudio era describir las experiencias de atención del personal de enfermería de las UCI durante la pandemia de COVID-19 en Turquía.

## DISEÑO Y MÉTODOS

### Entorno y Muestra

En este estudio cualitativo, fenomenológico-descriptivo y multicéntrico, los datos se recopilaron a través de la plataforma Zoom Cloud Meeting. Este estudio se llevó a cabo en hospitales públicos, que fueron los principales centros de la pandemia en Turquía entre abril de 2021 y septiembre de 2021. La muestra del estudio estuvo compuesta por enfermeras de UCI que trabajaron durante la pandemia de COVID-19, seleccionadas mediante el método de muestreo en bola de nieve. Se contactó a las enfermeras de UCI a través de las redes sociales. Además, se invitó a enfermeras de varios hospitales a participar a través de referencias de aquellas que ya habían aceptado participar. Las entrevistas fueron realizadas por la primera autora en turco, tras una presentación de sí misma y de los objetivos del estudio. Se incluyeron en el estudio las enfermeras que trabajaban activamente en las UCI durante la pandemia de COVID-19 y que aceptaron participar voluntariamente. Después de que un revisor externo confirmara la exactitud de los datos extraídos, las entrevistas y la codificación continuaron hasta alcanzar la saturación de datos. La saturación de datos se alcanzó después de la décima entrevista, cuando no surgieron nuevas propiedades o dimensiones. Se realizaron dos entrevistas adicionales para confirmar que no aparecían nuevos datos o códigos conceptuales. El equipo de investigación y un revisor externo determinaron colectivamente la saturación mediante una revisión continua. En este contexto, la muestra de la investigación incluyó a 12 enfermeras de UCI.

### Herramientas y Métodos de Recolección de Datos

Los investigadores utilizaron un formulario de entrevista para explorar los sentimientos y experiencias de los participantes. Diseñado para ser flexible y específico, el formulario contenía nueve preguntas abiertas. (Tabla 1).

**Tabla 1:** Experiencias asistenciales de las enfermeras que trabajan en la UCI COVID-19 Formulario – Çanakkale, Turquía, 2021.

**Preguntas**
1. ¿Podría explicar sus horas de trabajo en la UCI para COVID-19?
2. ¿Podría describer su experiencia en la atención de enfermería a un paciente consciente con COVID-19 en la UCI?
3. ¿Podría explicar su experiencia en la atención de enfermería a un paciente inconsciente con COVID-19 en la UCI?
4. ¿Podría describir sus experiencias emocionales relacionadas con el proceso de cuidados de enfermería a pacientes en la UCI?
5. ¿Podría explicar con más detalle las dificultades a las que se enfrentó durante el proceso de cuidados de enfermería a pacientes en la UCI?
6. ¿Podría explicar su proceso de comunicación y sus experiencias con los pacientes y sus familias?
7. ¿Podría describir sus experiencias en relación con la comunicación y el trabajo en equipo con sus compañeros?
8. ¿Podría compartir sus experiencias en relación con los principios éticos profesionales durante la atención a pacientes con COVID-19?
9. ¿Podría hablar sobre el evento más impactante durante el proceso de atención al paciente en la UCI por COVID-19?

El marco conceptual para las preguntas de la entrevista se definió mediante una revisión exhaustiva de la bibliografía sobre las experiencias de las enfermeras en la atención sanitaria durante la COVID-19^(1-8,11-14,19-26)^. Al crear el formulario de la entrevista, se tuvo cuidado de garantizar que las preguntas fueran claras y fáciles de entender, evitando al mismo tiempo las preguntas capciosas. El objetivo era obtener respuestas detalladas y precisas a preguntas abiertas, tanto directas como indirectas, y estructurar las preguntas en un flujo lógico y secuencial. El formulario de la entrevista comenzaba con preguntas generales y temas fáciles de responder. Posteriormente, fue revisado por un experto en enfermería con experiencia en investigación cualitativa en cuanto a contenido, alcance y lenguaje, y se realizaron las correcciones necesarias. Antes de iniciar las entrevistas del estudio principal, se llevaron a cabo dos estudios piloto con personas de la población objetivo que no fueron incluidas en el grupo de estudio final. Estos estudios piloto proporcionaron información sobre la claridad y la idoneidad de las preguntas, así como sugerencias para preguntas adicionales. Basándose en la información obtenida en estos estudios piloto, se revisó y finalizó el formulario de entrevista antes de comenzar las entrevistas principales. Se informó a los participantes en el estudio de que su información personal y sus respuestas serían confidenciales y que los datos solo se utilizarían con fines de investigación. Se realizaron entrevistas en profundidad a cada participante a través de la plataforma Zoom Cloud Meeting, utilizando un formulario de entrevista semiestructurado. Se realizaron grabaciones de audio y vídeo con el consentimiento de los participantes. Después de cada pregunta, se animaba a los participantes a dar más detalles sobre sus experiencias mediante preguntas de seguimiento como “¿Qué experiencias tuvo? ¿Qué pasó?”. Este enfoque tenía como objetivo obtener respuestas detalladas relacionadas con las preguntas. Para minimizar los posibles factores de confusión, los participantes realizaron las entrevistas solos en casa, en lugar de en el trabajo. Las entrevistas continuaron hasta que los participantes desearon detenerlas y se diseñaron para que parecieran una conversación. De media, las entrevistas duraron entre 50 y 75 minutos. Durante la recolección, categorización y análisis de los datos, se utilizaron inicialmente los nombres reales de los participantes. Sin embargo, tras completar la investigación, se asignó un número a cada participante para mantener la confidencialidad. Tras la transcripción de las grabaciones de audio en un proceso de decodificación mediante un programa informático, las transcripciones se leyeron varias veces para garantizar una comprensión profunda. Durante cada lectura, se desarrolló un marco conceptual para identificar posibles códigos. Las entrevistas fueron transcritas y codificadas por los investigadores, quienes analizaron y comunicaron los datos de acuerdo con los Criterios Consolidados para la Presentación de Informes de Investigación Cualitativa (COREQ) (Tabla 2).

**Tabla 2:** Criterios consolidados para la presentación de informes de investigación cualitativa (COREQ): lista de verificación de 32 puntos – Çanakkale, Turquía, 2021.

No	Elemento	Preguntasguía/Descripción	Informado en las páginasn.º
**Informado en las páginasn.º**			
**Características personales**			
1	Entrevistador/moderador	¿Qué autor o autores realizaron la entrevista o el grupo de discusión?	Métodos
2	Credenciales	¿Cuáles eran las credenciales del investigador?	Métodos
3	Ocupación	¿Cuál era su ocupación en el momento del estudio?	Métodos
4	Género	¿El investigador era hombre o mujer?	N/A
5	Experiencia y formación	¿Qué experiencia o formación tenía el investigador?	Métodos
**Relación con los participantes**			
6	Relación establecida	¿Se estableció una relación antes del inicio del estudio?	Métodos
7	Conocimiento que tenían los participantes del entrevistador	¿Qué sabían los participantes sobre el investigador?	Métodos
8	Características del entrevistador	¿Qué características se comunicaron sobre el entrevistador/facilitador? Por ejemplo, sesgos, suposiciones, motivos e intereses en el tema de investigación.	Métodos
**Ámbito 2: diseño del estudio**			
**Marco teórico**			
9	Orientación metodológica y teoría	¿Qué orientación metodológica se estableció como base del estudio?	Métodos
**Selección de participantes**			
10	Muestreo	¿Cómo se seleccionó a los participantes? Por ejemplo, intencional, por conveniencia, consecutivo, bola de nieve	Métodos
11	Método de acercamiento	¿Cómo se contactó a los participantes? Por ejemplo, en persona, por teléfono, por correo postal, por correo electrónico.	Métodos
12	Tamaño de la muestra	¿Cuántos participantes participaron en el estudio?	Métodos
13	No participación	¿Cuántas personas se negaron a participar o abandonaron? ¿Por qué motivos?	N/A
**Entorno**			
14	Entorno de recopilación de datos	¿Dónde se recopilaron los datos? Por ejemplo, en el hogar, en la clínica, en el lugar de trabajo.	Métodos
15	Presencia de personas no participantes	¿Había alguien más presente además de los participantes y los investigadores?	Métodos
16	Descripción de la muestra	¿Cuáles son las características importantes de la muestra? Por ejemplo, datos demográficos, fecha	Métodos
**Recolección de datos**			
17	Guía de entrevista	¿Los autores proporcionaron preguntas, indicaciones y guías? ¿Se realizó una prueba piloto?	Métodos
18	Entrevistas repetidas	¿Se realizaron entrevistas repetidas? En caso afirmativo, ¿cuántas?	N/A
19	Grabación audiovisual	¿Se utilizó grabación audiovisual para recopilar los datos?	Métodos
20	Notas de campo	¿Se tomaron notas de campo durante y/o después de la entrevista o el grupo focal?	Métodos
21	Duración	¿Cuálfue la duración de las entrevistas o del grupo focal?	Métodos
22	Saturación de datos	¿Se discutió la saturación de datos?	Métodos
23	Devolución de las transcripciones	¿Se devolvieron las transcripciones a los participantes para que las comentaran o corrigieran?	Métodos
**Ámbito 3: análisis y resultados**			
**Análisis de datos**			
24	Número de codificadores de datos	¿Cuántos codificadores de datos codificaron los datos? El proceso de codificación fue llevado a cabo por ambos investigadores conjuntamente.	Resultados
25	Descripción del árbol de codificación	¿Los autores proporcionaron una descripción del árbol de codificación?	Resultados
26	Derivación de lostemas	¿Se identificaron los temas de antemano o se derivaron de los datos?	Resultados
27	Software	¿Qué software, si procede, se utilizó para gestionar los datos?	Métodos
28	Verificación de los participantes	¿Los participantes proporcionaron comentarios sobre los resultados?	Conclusión
**Informes**			
29	Citas presentadas	¿Se presentaron citas de los participantes para ilustrar los temas/resultados? ¿Se identificó cada cita? Por ejemplo, número de participante.	Resultados
30	Datos y conclusiones coherentes	¿Hubo coherencia entre los datos presentados y los hallazgos?	Relación con los conocimientos existentes
31	Claridad de los temas principales	¿Se presentaron claramente los temas principales en los resultados?	Resultados
32	Claridad de los temas secundarios	¿Hay una descripción de casos diversos o un debate sobre los temas secundarios?	Discusión

### Análisis de Datos

Durante el proceso de entrevistas, se obtuvo el consentimiento verbal de los participantes y se realizaron grabaciones de video y audio. La transcripción de estas grabaciones se llevó a cabo en archivos de Microsoft Word después de las entrevistas. Tras la transcripción de cada entrevista, un miembro del equipo de investigación nativo de habla turca realizó una traducción inversa. A continuación, cada traducción fue traducida de nuevo al inglés por un profesor de inglés. Se compararon y revisaron la primera y la segunda traducción al inglés. Por último, para garantizar la precisión de la traducción, el contenido fue revisado y aprobado por un revisor externo con dominio del turco y el inglés y con experiencia en investigación cualitativa. Cada texto de la entrevista se leyó varias veces, palabra por palabra, frase por frase y párrafo por párrafo. Inicialmente, se realizaron varias entrevistas y se llevó a cabo la codificación. A continuación, se clasificaron de forma preliminar los códigos extraídos. La transcripción y la codificación fueron realizadas por el primer y el segundo autor. Para verificar la exactitud de los datos extraídos, las entrevistas, el proceso de codificación y los datos clasificados fueron revisados por un revisor externo especializado en estudios cualitativos. Una vez que el revisor externo confirmó la exactitud de los datos extraídos, el equipo de investigación continuó con las entrevistas y la codificación hasta alcanzar la saturación de datos. En la etapa final, tras alcanzar la saturación de datos, el equipo de investigación revisó los códigos extraídos. Se eliminaron los códigos duplicados y se derivaron de forma inductiva categorías y subcategorías a partir de los datos brutos iniciales. Para garantizar aún más la exactitud de los datos, el revisor externo volvió a examinar los datos clasificados y se modificaron los resultados en función de sus comentarios. El análisis de datos se realizó utilizando MAXQDA 20. Se emplearon análisis descriptivos, el modelo de teoría del código, el modelo jerárquico de código-subcódigo y análisis comparativos y relacionales. En la fase de presentación de informes, se explicaron las categorías, se proporcionaron descripciones y se interpretaron los resultados.

Los resultados de la investigación se respaldaron con citas directas de los participantes. Para garantizar la coherencia de los resultados, se examinó la coherencia de cada categoría y su contribución a un todo significativo. Los datos para recolectar se informaron en detalle, garantizando la transparencia desde la recolección de datos hasta la presentación de los resultados. Las generalizaciones realizadas al final del estudio se explicaron como dentro de las limitaciones de los resultados y específicas del grupo estudiado. Los datos de las entrevistas se presentaron sin interpretación. Un experto independiente revisó y confirmó la codificación, las categorías y el análisis. El proceso de categorización comenzó con la técnica in vivo, luego se ajustó en función de las preguntas de investigación y, finalmente, se perfeccionó utilizando conceptos teóricos. Los resultados se compararon con otros estudios y se respaldaron con bibliografía relevante. En el proceso de presentación de informes, se explicaron las categorías, se describieron las relaciones y se interpretaron los resultados. Se logró una comprensión holística mediante la identificación de las relaciones de causa y efecto y la extracción de conclusiones a partir de los resultados. Durante la fase de presentación de informes, los investigadores volvieron a leer los registros y seleccionaron citas apropiadas para incluir en el informe^(5,6)^.

### Aprobaciones Éticas

El Ministerio de Salud de Turquía (aprobación 19T14-01-58 de fecha 09/05/2020) y el Comité de Ética en Investigación de la Universidad Çanakkale Onsekiz Mart concedieron la aprobación ética para este estudio (aprobación inicial del ensayo 2000184619 de fecha 06/06/2020). Los participantes dieron su consentimiento verbal para la grabación de audio y vídeo.

## RESULTADOS

Los perfiles de los participantes se presentanen la tabla 3.

**Tabla 3:** Perfil de los participantes – Çanakkale, Turquía, 2021.

Participantes	Edad	Sexo	Estado civil Estado	Educación	¿Con quién vive?	¿Fumas?	Enfermedades crónicas	Experiencia en la UCI (año)	Resultados de la prueba PCR
**P1**	22–30	F	Única	Licenciatura	Familia	No	No	1–5	Negativo
**P2**	22–30	F	Único	Licenciatura	Solo	No	No	1–5	Negativo
**P3**	≥31	F	Casado	Máster	Familia	No	No	≥ 6	Positivo
**P4**	22–30	M	Casado	Licenciatura en Ciencias	Familia	No	No	< 1	Positivo
**P5**	≥31	F	Casada	Licenciado	Familia	Sí	No	≥ 6	Negativo
**P6**	≥31	F	Casado	Licenciado	Familia	No	Sí	1–5	Negativo
**P7**	≥31	F	Casado	Máster	Familia	Sí	No	≥ 6	Negativo
**P8**	22–30	F	Soltero	Máster	Familia	No	No	≥ 6	Negativo
**P9**	22–30	M	Soltero	Licenciatura	Solo	Sí	No	1–5	Positivo
**P10**	22–30	F	Soltero	Licenciatura	Solo	No	No	1–5	Positivo
**P11**	22–30	F	Soltero	Licenciatura	Solo	No	Sí	< 1	Negativo
**P12**	≥31	F	Casada	Licenciado	Familia	No	No	1–5	Negativo

Se obtuvieron un total de 851 códigos primitivos. Se identificaron 13 subcódigos, 47 códigos, 12 categorías y cuatro temas. (a) Categorías del tema “Enfoque de atención integral”: barreras, comunicación y atención espiritual. (b) Categorías del tema “Gestión de procesos”: precauciones, retos, adaptación y procesos de tratamiento. (c) Categorías del tema “Efectos físicos y psicológicos”: efectos físicos y efectos psicológicos. (d) Categorías del tema “Dilema ético”: comportamientos éticos, comportamientos no éticos y principios de trabajo (Tabla 4).

**Tabla 4:** Proceso de resumen de datos – Çanakkale, Turquía, 2021.

Marco	Temas	Categorías	Códigos	Subcódigos
**Viaje a la UCI Durante la pandemia de COVID-19 c**	**Enfoque de atención integral**	Barreras	Sustitución continua de EPP Suministro de materiales Fatiga Aplazamiento de las necesidades básicas La prolongada duración de los cuidados	
		Comunicación	Comunicación con lospacientes Comunicación con la familia del paciente	
		Atención espiritual	Apoyo Moral	
	**Gestión de procesos**	Precauciones	Precauciones individuales Precauciones institucionales	
		Retos	Ignorancia Psicología del paciente Pronóstico de la enfermedad Incertidumbre EPP Riesgo de transmisión/infección Carga de trabajo Falta de materiales Falta de personal Conflictos en el equipo	
		Adaptación	Apoyo del equipo Coordinación del equipo Satisfacción Contribución al proceso Enfoque corporativo	
		Proceso de tratamiento	Perfil del paciente Plan de tratamiento Complicaciones Habilidades adquiridas Ánimo	
	**Efectos físicos y psicológicos**	Efectos físicos	Deterioro de la salud Dolor de cabeza Agotamiento Sudoración	
		Efectos psicológicos	Efectos negativos	Tristeza Burnout Ansiedad/miedo Estrés Impotencia Malestar Desesperanza Desilusión
			Efectos positivos	Adaptación Felicidad Dedicación Realización espiritual Motivación
	**Dilema ético**	Comportamientos éticos	Conciencia Cuidado igualitario Responsabilidad	
		Comportamiento poco ético	Discriminación de pacientes Falta de atención	
		Principios de trabajo	Integridad Conciencia Responsabilidad No discriminación Beneficencia	

### (A) Enfoque Holístico/Integral de la Atención

La categoría de barreras se ha examinado en los códigos de sustitución continua de EPP, suministro de materiales, fatiga, aplazamiento de necesidades básicas y duración prolongada de la atención. La categoría de comunicación se ha examinado en los códigos de comunicación con los pacientes y con la familia del paciente, y la categoría de atención espiritual se ha examinado en los códigos de apoyo y moral.

Muchos participantes informaron de que el uso de EPP prolongaba la duración de la atención al paciente. Mencionaron que el elevado consumo de EPP y otros suministros hacía que la adquisición de estos materiales fuera un proceso que requería mucho tiempo. Los participantes también destacaron que prestar atención sanitaria con EPP provocaba fatiga, sudoración excesiva y cambios frecuentes de uniforme. Algunos participantes señalaron que posponían necesidades básicas como comer y beber agua debido a la preocupación por el uso del EPP y al miedo a la infección. Los participantes también afirmaron que su carga de trabajo aumentó debido a la falta de personal y al aislamiento. Algunos participantes informaron de que los pacientes conscientes permanecían en contacto constante con ellos debido a su incapacidad para comunicarse con sus familias. Estos pacientes a menudo experimentaban ansiedad y miedo a la muerte, lo que les llevaba a interactuar frecuentemente con las enfermeras. Muchos participantes afirmaron que prestaban apoyo a los pacientes de diversas maneras.

“Los materiales consumibles, como los EPP, se utilizan en grandes cantidades y su adquisición lleva mucho tiempo”. (P8)

“Estamos siempre con los pacientes. A veces quieren cogernos de la mano. El paciente, psicológicamente, quiere que estés ahí. Te dicen: ‘No te vayas’”. (P9)

“La zona se calienta mucho. Te ves obligado a sudar. ¿Cuántas veces te cambias de ropa? Te entra sed, pero no puedes beber agua, y cuando te tomas un descanso para beber, tienes que ir al baño durante la atención”. (P11)

“La atención al paciente varía según su estado. Si el paciente está intubado, la atención lleva mucho tiempo. Cuando hacemos un descanso, cambiamos constantemente el EPP y los uniformes”. (P12)

Cuando se evaluó el tema del enfoque de atención holística/integral basándose en las opiniones de los participantes, se determinó que sus opiniones se centraban principalmente en el apoyo. Además, cuando se evaluó este tema según el estado civil de los participantes, las enfermeras solteras expresaron opiniones más intensas sobre la comunicación con los pacientes en comparación con las enfermeras casadas.

### (B) Gestión de Procesos

La categoría de precauciones se ha examinado bajo los códigos de precauciones individuales y precauciones institucionales, la categoría de retos se ha examinado bajo los códigos de ignorancia, psicología del paciente, pronóstico de la enfermedad, incertidumbre, EPP, riesgo de transmisión/infección, carga de trabajo, falta de materiales, falta de personal y conflicto de equipo. La categoría de adaptación se ha examinado bajo los códigos de apoyo del equipo, coordinación del equipo, satisfacción, contribución al proceso y enfoque corporativo, y la categoría de proceso de tratamiento se ha examinado bajo los códigos de perfil del paciente, plan de tratamiento, complicaciones, habilidades obtenidas y ánimo.

En la categoría de precauciones, los participantes compartieron sus experiencias con respecto al uso de EPP y la fatiga causada por llevarlo puesto. En la categoría de retos, el problema más frecuente identificado por los participantes fue la carga de trabajo. Los participantes atribuyeron el aumento de su carga de trabajo a la falta de personal. También afirmaron que a menudo se les exigía realizar tareas propias de otras disciplinas por diversas razones, lo que aumentaba aún más su carga de trabajo. Muchos participantes informaron de dificultades para colaborar tanto con las enfermeras como con otros miembros del equipo.

“No podía cambiar la posición de mi paciente cada dos horas. ¿Por qué? Porque hay falta de personal. No puedo hacerlo solo”. (P2)

“Nuestros procesos de atención suelen llevar mucho tiempo. Lo llamamos el agujero negro. Nos quedamos aquí al menos entre tres y cuatro horas”. (P3)

“Lo que más me cuesta es relacionado con la visión. Porque tengo mis propias gafas, por desgracia. Cuando me pongo las gafas protectoras y la pantalla facial, al cabo de media hora se empañan. Como no puedo salir de la zona, tengo que seguir así”. (P6)“Aunque teníamos pacientes a los que atendíamos, incluyendo cuidados bucales, teníamos muchos conflictos cuando los médicos no entraban en la UCI, observaban desde lejos o daban órdenes verbales por teléfono”. (P7)

En la categoría de adaptación, el código más mencionado fue la contribución al proceso. Los participantes mencionaron que trabajar en consonancia con el equipo a veces ayudaba a reducir la carga de trabajo. Además, algunos participantes expresaron sentimientos positivos y satisfacción durante la pandemia. Reflexionaron sobre cómo trabajar en la UCI de COVID-19 enriqueció su experiencia profesional y les permitió ser testigos de un proceso histórico.

“Intentamos turnarnos en la atención al paciente. Pero a veces, todos tenemos que involucrarnos en la atención. Tenemos atención tres veces al día y dedicamos aproximadamente 16 horas del turno de 24 horas a los pacientes”. (P7)

“El paciente dijo: ‘Nunca os olvidaré. Si no me hubieran proporcionado este apoyo psicológico, no sé si habría podido salir de aquí así’”. (P8)

En la categoría del proceso de tratamiento, los participantes indicaron que muchos pacientes eran jóvenes y no padecían enfermedades crónicas. Los participantes también informaron de que a menudo se producían lesiones por presión debido a la posición de los pacientes y al equipo utilizado. Algunos pacientes tenían dificultades para tolerar la posición boca abajo. Además, algunos participantes afirmaron que adquirieron una valiosa experiencia durante el trabajo y la aplicaron al tratamiento de otros pacientes.

“Intentamos prevenir las lesiones por presión cambiando constantemente la posición de los pacientes que estaban boca abajo en las zonas del pecho, la barbilla y la frente”. (P1)

“El estado general de los pacientes puede deteriorarse muy rápidamente. Un paciente consciente puede sufrir una parada cardíaca repentina. Los pacientes conscientes también tienen miedo. Hablas con ellos. Te conviertes en su esperanza”. (P12)

Cuando se evaluó el tema de la gestión de procesos basándose en las opiniones de los participantes, se determinó que sus opiniones se centraban principalmente en la duración de los cuidados. Además, cuando se evaluó este tema según la experiencia laboral de los participantes en la UCI, los participantes con entre 1 y 5 años de experiencia laboral en la UCI expresaron opiniones más intensas con respecto al EPP.

### (C) Efectos Físicos y Psicológicos

La categoría de efectos físicos se examinó bajo los códigos de deterioro de la salud, dolor de cabeza, agotamiento y sudoración.

Los participantes afirmaron que el EPP que utilizaban les provocaba dolores de cabeza y sudoración excesiva. Algunos participantes informaron de que habían experimentado enfermedades y pérdida de cabello, mientras que otros expresaron que se sentían físicamente agotados durante su trabajo.

“Antes se me caía muy poco el pelo. Ahora, debido al exceso de trabajo, se me cae en exceso. También he empezado a despertarme gritando por las noches. Además, colocar a los pacientes me agotaba físicamente”. (P4)

“Llevar varias capas de mascarillas me provocaba fuertes dolores de cabeza”. (P5)

“Sudo constantemente debido al uso del EPP, pero también tengo que cuidar al paciente”. (P12)

La categoría de efectos psicológicos se ha examinado en función de los efectos negativos y positivos. El código de efectos negativos se ha examinado en función de los subcódigos de tristeza, agotamiento, ansiedad/miedo, estrés, impotencia, malestar, desesperanza y desilusión (Tabla 4). En el código de efectos negativos, la emoción expresada con mayor intensidad fue la tristeza. Los participantes describieron el impacto emocional de las frecuentes pérdidas de pacientes, en particular de los más jóvenes, así como la carga que supone el exceso de trabajo. Muchos participantes informaron sentirse angustiados debido a la distribución injusta de las tareas y su pesada carga de trabajo. También compartieron sentimientos de agotamiento y miedo, en particular en relación con el riesgo de contraer la COVID-19 o de transmitirla a sus familias. Además, algunos participantes expresaron su frustración por la insuficiencia de los EPP, que les hacía sentir insuficientemente protegidos, y sentimientos de desesperanza cuando los pacientes no se recuperaban.

El código de efectos positivos se ha examinado en los subcódigos de adaptación, felicidad, dedicación, plenitud espiritual y motivación. Sin embargo, los participantes también mencionaron momentos de felicidad y motivación, especialmente cuando los pacientes se recuperaban, cuando eran testigos de la dedicación de sus colegas y cuando experimentaban plenitud espiritual a través de su trabajo. Muchos participantes expresaron su orgullo por su compromiso con su profesión durante la pandemia.

“En este momento, es el peor período que he vivido en mi profesión... Estamos indefensos. Tenemos miedo. Estamos tristes... Las dificultades a las que nos enfrentamos en la atención debido a la escasez de personal son importantes. La motivación disminuye. Te vuelves infeliz”. (P3)

“La enfermería me da mucho placer. Cuando un paciente me da las gracias, me motiva increíblemente”. (P6)

“En la UCI, toda la atención al paciente, su apoyo psicológico, sus necesidades físicas y la carga general recaen sobre las enfermeras. El paciente no tiene a nadie más en quien confiar. Depende completamente de ti, como un bebé. También experimentamos satisfacción a través de esta responsabilidad”. (P11)

Cuando se evaluó el tema de los efectos físicos y psicológicos basándose en las opiniones de los participantes, la tristeza y el agotamiento fueron las opiniones más comunes. Además, cuando se evaluó este tema según los resultados de las pruebas de COVID-19 de los participantes, los participantes con resultados negativos experimentaron emociones negativas más intensas en comparación con los que dieron positivo.

### (D) Dilema Ético

La categoría de comportamientos éticos se ha examinado bajo los códigos de conciencia, igualdad de atención y responsabilidad; la categoría de comportamientos no éticos se ha examinado bajo los códigos de discriminación de pacientes y falta de atención, y la categoría de principios de trabajo se ha examinado bajo los códigos de integridad, conciencia, responsabilidad, no discriminación y beneficencia.

En este tema, los participantes expresaron opiniones firmes sobre el código de conciencia, haciendo hincapié en sus esfuerzos por proporcionar una atención equitativa a todos los pacientes sin discriminación. Además, debatieron sobre sus experiencias morales y emocionales durante el proceso de atención al paciente. En la categoría de comportamientos poco éticos, los participantes mencionaron varios factores que contribuían a una atención inadecuada, entre ellos la falta de personal, la insuficiencia de EPP, el hecho de que los médicos no entraran en la UCI y que algunas enfermeras se negaran a prestar atención debido al riesgo de infección. En la categoría de principios de trabajo, el código más destacado fue el de la responsabilidad. Muchos participantes afirmaron que defendían sistemáticamente el principio de proporcionar una atención igualitaria y justa a todos los pacientes, independientemente de las circunstancias.

“Presto atención a proporcionar una atención igualitaria y justa a todos los pacientes”. (P10)

“No puedo decir con la conciencia tranquila que he completado toda la atención a mis pacientes. No pude proporcionar la posición necesaria a mi paciente cuando lo necesitaba. No pude proporcionar la atención suficiente para prevenir lesiones por presión o para el desarrollo de los pacientes”. (P3)

“Los pacientes se vieron privados de una atención adecuada. No pudimos proporcionarles una atención suficiente”. (P5)

“Los pacientes no eligieron tener esta enfermedad a sabiendas. No debería proporcionarles una atención inadecuada. Independientemente de la raza, el sexo o la edad del paciente, debería proporcionarles atención”. (P10)

Cuando se evaluó el tema del dilema ético basándose en las opiniones de los participantes, estos expresaron predominantemente su preocupación por la falta de cuidados adecuados. Además, cuando se evaluó este tema según la edad de los participantes, los menores de 30 años expresaron opiniones más intensas sobre la conciencia y la igualdad en los cuidados, mientras que los mayores de 31 años expresaron una mayor preocupación por la falta de cuidados adecuados.

Los análisis relacionales surgieron de la convergencia de las opiniones de los participantes. Los participantes no solo compartieron sus retos, sino que también debatieron otras cuestiones relacionadas, como la escasez de EPP, las precauciones, la carga de trabajo, la duración de la atención y el deterioro de la salud (Figura 1).

**Figura 1:** Mapa de códigos – Çanakkale, Turquía, 2021.

Un participante resumió la situación de la siguiente manera:

“Para el proceso de atención y tratamiento de cada paciente, nos ponemos la bata, el gorro, la mascarilla N95 y la mascarilla quirúrgica, la visera, los cubrezapatos... El proceso de atención dura al menos una hora y media para un paciente intubado. Cuidado bucal, aspiración del tubo endotraqueal, cambio de ligadura del tubo, colocación en posición prona, evaluación de lesiones por presión, etc. En el caso de algunos pacientes, la atención dura tres horas. Los cambios de posición se realizan cada dos horas. También proporcionamos apoyo psicológico y moral a los pacientes conscientes. Todo es muy difícil; nuestra salud física y mental se ha deteriorado”(P4).

## DISCUSIÓN

Los datos del estudio se recolectaron entre abril y septiembre de 2021, un periodo que corresponde a la fase posterior a la vacunación, pero aún de alto riesgo de la pandemia de COVID-19 en Turquía. Durante este tiempo, las UCI siguieron sometidas a una presión considerable debido a las fluctuaciones en las tasas de infección y al tratamiento continuo de pacientes en estado crítico. Aunque la fase inicial de incertidumbre aguda había remitido, persistían las preocupaciones sobre las variantes del virus, la escasez de EPP y las cargas de trabajo prolongadas. Por lo tanto, el momento de la recolección de datos refleja una fase caracterizada por una carga clínica sostenida, un aumento del cansancio profesional y una adaptación gradual a las condiciones de crisis a largo plazo. En la literatura, los profesionales sanitarios se enfrentaron a numerosos retos, entre ellos la exposición a patógenos, el aumento de la carga de trabajo y la prolongación de la jornada laboral, todo lo cual contribuyó a la fatiga, el agotamiento profesional y el malestar psicológico^(1,2)^. El cuidado de los pacientes con COVID-19 fue especialmente exigente para las enfermeras, con efectos documentados como la disminución del apetito, los trastornos del sueño y la fatiga. Además, se informó de que el uso de EPP causaba molestias físicas, restringía los movimientos, provocaba sensación de asfixia, inducía sudoración excesiva, retrasaba las necesidades básicas y provocaba deshidratación y estreñimiento^(3–7)^.

En nuestro estudio, los participantes describieron de manera similar el trabajo con EPP como un reto físico y mental. Informaron de sesiones de cuidados de enfermería prolongadas sin descanso adecuado, lo que provocaba fatiga. Otras preocupaciones adicionales fueron la sudoración excesiva, los cambios frecuentes de uniforme y el aplazamiento de las necesidades físicas básicas. Los participantes también señalaron que la carga de trabajo aumentó debido a la escasez de personal y a la renuencia de algunos miembros del personal a entrar en las áreas de la UCI por temor a la infección. La literatura indica que factores como la reducción de personal en la UCI, los intentos de prestar el mismo nivel de atención con menos personal y las exigencias del uso de EPP y el cumplimiento de las medidas de aislamiento repercuten negativamente en la atención integral del paciente^(3)^.

Durante este estudio, los participantes hicieron hincapié en la importancia de un enfoque holístico y integral de la atención al paciente, dando prioridad a las necesidades psicológicas junto con el tratamiento físico. Los participantes informaron de que el aislamiento de los pacientes en la UCI, la falta de contacto con la familia y la gravedad de la enfermedad aumentaban la ansiedad de los pacientes. Para mitigar esto, los participantes describieron cómo proporcionaban apoyo emocional sosteniendo las manos de los pacientes, hablando con ellos y ofreciéndoles ánimo moral para reducir su miedo a la muerte y aliviar la ansiedad. Como único vínculo entre los pacientes y sus familias, las enfermeras desempeñaron un papel fundamental a la hora de atender las necesidades emocionales de los pacientes. En consonancia con estos hallazgos, la bibliografía destaca que el apoyo espiritual proporcionado por el personal sanitario es crucial para los pacientes con COVID-19, y que las enfermeras a menudo se ponen en riesgo para fortalecer las relaciones entre ellas y los pacientes a pesar del entorno estresante^(2,15)^.

Otro estudio informó que las enfermeras, como primer punto de contacto con los pacientes, a menudo les brindaban calidez y cuidados a pesar de su propio cansancio y ansiedad, tratando a los pacientes como si fueran miembros de su familia^(5)^. De manera similar, en nuestro estudio, los participantes se comunicaban con frecuencia con los pacientes conscientes, ayudándoles a aliviar sus miedos e incorporando la educación del paciente en sus procesos de atención. Sin embargo, debido a las limitaciones de la infraestructura tecnológica de la UCI, los participantes no podían facilitar la comunicación directa entre los pacientes y sus familias. En su lugar, se encargaron de informar a las familias sobre el estado de los pacientes.

En la presente investigación, los participantes destacaron con frecuencia la falta de EPP como un reto importante durante el proceso de atención. Señalaron que la ausencia de EPP y personal adecuados aumentaba tanto la duración de la atención al paciente como su carga de trabajo general. Los participantes también informaron de que algunos miembros del equipo evitaban cumplir con sus obligaciones al no entrar en la UCI por miedo a infectarse. Además, los participantes expresaron su preocupación por contraer el virus ellos mismos y poder contagiar a sus seres queridos. La incertidumbre sobre la duración de la pandemia y el mal pronóstico de muchos pacientes agravaban aún más su estrés y su carga emocional. En la literatura, las enfermeras han informado de problemas como la falta de EPP, medidas de protección inadecuadas y directrices insuficientes sobre el uso de EPP, además del aumento de la carga de trabajo debido a la escasez de personal. Aunque los pacientes con COVID-19 presentan una amplia gama de afecciones clínicas, los estudios han documentado que incluso los pacientes jóvenes sin comorbilidades preexistentes pueden experimentar un deterioro clínico repentino, que a menudo requiere el uso de ventiladores mecánicos^(3,6,27)^. En este trabajo, los participantes observaron que los pacientes sin enfermedades crónicas podían experimentar un rápido deterioro de su estado. La frecuente mención de los perfiles genéticos de los pacientes y la mortalidad por parte de los participantes sugiere el posible desarrollo de fatiga por compasión en este contexto. A pesar de estos retos, algunos participantes señalaron que trabajar en condiciones tan exigentes les proporcionó experiencias profesionales únicas y fomentó el trabajo en equipo. De manera coherente, la literatura indica que las enfermeras informaron de mejoras en sus prácticas de aislamiento, habilidades de comunicación y crecimiento profesional general durante la pandemia^(28)^.

Durante la presente investigación, los participantes informaron haber experimentado importantes desafíos físicos y psicológicos, como dolores de cabeza, fatiga, estrés y ansiedad. Estos desafíos surgieron del uso prolongado de EPP, las cargas de trabajo y el riesgo de transmisión de enfermedades durante el proceso de atención. En consonancia con estos hallazgos, investigaciones anteriores han demostrado que las enfermeras experimentaron niveles notablemente altos de fatiga ocupacional durante la pandemia^(14)^. El uso de EPP se ha asociado con diversas molestias físicas, como dolores de cabeza, dificultades respiratorias, ataques de pánico, dermatitis y reacciones alérgicas^(4,7)^. Un estudio informó que el uso de EPP restringía la movilidad, elevaba la temperatura corporal, aumentaba la sudoración y creaba una sensación de asfixia^(5)^. En otro estudio, los participantes informaron de una disminución del apetito, fatiga y dificultad para dormir como experiencias comunes^(8)^. La fatiga física se relacionó con el proceso de aislamiento y el uso de EPP, ya que su uso continuo provocaba sudoración excesiva, deshidratación y agotamiento durante los periodos de atención prolongados^(9)^.

Los efectos psicológicos también se han documentado ampliamente en la literatura. Las enfermeras informaron con frecuencia de miedo a contraer el virus, reticencia a interactuar con los familiares y sentimientos de inseguridad. Estas experiencias se vieron agravadas por el estrés, la ansiedad, la depresión, la confusión, la irritabilidad y la inquietud. La incapacidad de evitar el rápido deterioro de los pacientes y las frecuentes pérdidas de pacientes provocaron fatiga por compasión, junto con sentimientos de tristeza, ira, culpa, pesimismo, desesperanza y agotamiento emocional. Las enfermeras también describieron la pérdida de motivación y la sensación de falta de sentido en su trabajo^(6,8)^. En un estudio, los participantes expresaron una intensa ansiedad al cuidar a pacientes en áreas de aislamiento, así como el miedo a infectarse y a infectar a sus familias. También se informó con frecuencia de la preocupación por tener que atender a un gran número de pacientes con personal insuficiente^(15)^. Ser testigos del sufrimiento de los pacientes, sentirse impotentes para aliviarlo y observar la muerte de pacientes jóvenes resultó especialmente angustioso para las enfermeras, y algunas describieron estas experiencias como insoportables^(5)^.

Nuestros hallazgos corroboran estas observaciones. En este estudio, los participantes experimentaron efectos físicos y psicológicos graves a lo largo del proceso de atención. A pesar de estos retos, los participantes describieron la recuperación de los pacientes como una fuente de inmensa alegría, a menudo comparándola con un milagro. Señalaron que la gratitud expresada por los pacientes en recuperación servía como un fuerte factor de motivación, inspirándoles a esforzarse por ofrecer la mejor atención posible. Del mismo modo, la literatura destaca que las enfermeras, a pesar de su fatiga y sus dificultades personales, suelen tratar a sus pacientes con el mismo cuidado y atención que darían a un familiar, sin esperar nada a cambio^(5)^.

Nuestro estudio reveló que la mayoría de los participantes informaron de que no podían proporcionar una atención adecuada debido a factores como la escasez de personal y la falta de materiales. Del mismo modo, un estudio descubrió que la reducción de personal aumentaba la carga de trabajo de las enfermeras, mientras que las enfermeras recién asignadas tenían dificultades para prestar una atención de calidad debido a sus conocimientos insuficientes^(3)^. Los participantes en el presente estudio también describieron sentirse abandonados en la UCI, afirmando que la atención que prestaban a los pacientes se limitaba a lo que podían manejar, lo que les causaba un importante estrés moral. Un estudio informó de que, aunque las enfermeras pasaban largos periodos con los pacientes durante el proceso de atención y tratamiento, los médicos solían estar ausentes, no se respetaban los principios de igualdad y las enfermeras tenían que realizar con frecuencia procedimientos que normalmente llevaban a cabo los médicos^(11)^. Del mismo modo, los resultados revelaron que la calidad de la atención en las UCI de COVID-19 disminuyó debido al aumento de la carga de trabajo y la reducción de personal. La literatura indica además que la combinación del riesgo de transmisión, el cese del tratamiento de los pacientes en estado crítico con alto riesgo de mortalidad y la escasez de personal y de equipo de protección personal a menudo obliga a las enfermeras a proporcionar una atención incompleta^(12,13)^. Esto incluye comprometer la seguridad de los pacientes y la calidad de la atención, así como descuidar las actividades de enfermería rutinarias, como el cuidado bucal, la prevención de lesiones por presión, la movilización y la detección del delirio en la UCI^(13)^.

En nuestro estudio, algunos participantes intentaron permanecer más tiempo con los pacientes para satisfacer todas sus necesidades, mientras que otros se sintieron culpables por no poder proporcionar una atención adecuada. Los participantes también informaron de que en múltiples ocasiones tuvieron que elegir entre las necesidades de los pacientes y su propia seguridad. No obstante, los participantes hicieron hincapié en que, para ellos, todos los pacientes son iguales y que se esfuerzan por garantizar que el tratamiento, la atención y las instalaciones sean lo más equitativos posible. La literatura destaca que las enfermeras a menudo se sienten incapaces de proporcionar una atención adecuada y, en ocasiones, se enfrentan a dilemas éticos en los que deben elegir entre dar prioridad a su propio bienestar o al de sus pacientes. Curiosamente, muchas enfermeras no reconocen estos dilemas como cuestiones éticas^(5,29)^. En nuestro estudio, los participantes informaron de que aplicaban los principios de beneficencia, no maleficencia y justicia en sus procesos de atención. Sin embargo, a pesar de que el objetivo fundamental de la enfermería es aliviar el sufrimiento y proporcionar una atención de calidad, varios participantes reconocieron que no podían prestar una atención adecuada debido a los diversos retos que se les presentaban durante el proceso de atención. Algunos participantes también informaron que trabajar en la UCI de COVID-19 era beneficioso desde el punto de vista profesional, ya que les proporcionaba oportunidades de aprendizaje únicas y nuevas perspectivas. Del mismo modo, la literatura sugiere que la pandemia ofreció un crecimiento profesional inesperado a muchas enfermeras, incluyendo una comprensión más profunda de su profesión, una mayor resiliencia, experiencia en el manejo de pacientes con COVID-19 y mejores habilidades de gestión de crisis^(28,30)^.

El contexto temporal de la recolección de datos también influyó en las experiencias y percepciones de riesgo comunicadas por las enfermeras de la UCI. La realización de entrevistas durante la fase media-tardía de la pandemia significó que los participantes ya habían desarrollado mecanismos de adaptación y una mayor familiaridad con los protocolos de tratamiento de la COVID-19. Sin embargo, también informaron de fatiga acumulada y agotamiento emocional por la exposición prolongada a situaciones de crisis. A diferencia de los estudios realizados durante la fase inicial del brote, en los que predominaban el miedo a la infección y la incertidumbre^(2,6,15)^, los participantes en este estudio describieron una compleja mezcla de tensión crónica, angustia moral y resistencia profesional. Este matiz contextual es esencial para interpretar los resultados, ya que sitúa las experiencias de las enfermeras de las UCI turcas en una trayectoria dinámica de adaptación, resiliencia y estrés laboral continuo.

Los resultados de este estudio subrayan la necesidad de políticas institucionales que den prioridad al bienestar psicológico, la seguridad física y la sostenibilidad profesional de las enfermeras de las UCI, especialmente en situaciones de crisis como las pandemias. Las instituciones deben adoptar un enfoque proactivo y multidimensional que integre el apoyo psicológico continuo, las sesiones informativas estructuradas y las políticas de dotación de personal flexibles para fomentar la resiliencia y mejorar la calidad de la atención. Dada la tensión emocional, la tristeza y el agotamiento documentados que experimentan las enfermeras de las UCI, los hospitales deben implementar sistemas de apoyo psicológico permanentes y accesibles. Estos podrían incluir revisiones periódicas de la salud mental, servicios de asesoramiento y grupos de apoyo entre pares dirigidos por psicólogos o enfermeras de salud mental capacitados. La integración de la promoción de la salud mental en la práctica clínica diaria a través de talleres de manejo del estrés, capacitación en resiliencia e intervenciones basadas en la atención plena puede reducir las secuelas psicológicas a largo plazo y aumentar la satisfacción profesional.

Las sesiones estructuradas de análisis tras incidentes críticos, pérdidas de pacientes o turnos prolongados de alta intensidad permiten a las enfermeras expresar sus experiencias, procesar el estrés moral y extraer un significado compartido de su trabajo. Estas sesiones no solo mitigan el agotamiento emocional, sino que también contribuyen a una cultura de seguridad psicológica y cohesión del equipo. Las reuniones periódicas de reflexión también pueden ayudar a identificar retos sistémicos, fomentar el aprendizaje mutuo y fundamentar la toma de decisiones de la dirección. Los resultados ponen de relieve cómo la escasez de personal, la prolongación de la duración de los cuidados y la excesiva carga de trabajo comprometieron la seguridad tanto del personal de enfermería como de los pacientes. Las instituciones deben desarrollar modelos de dotación de personal flexibles que tengan en cuenta los aumentos repentinos del volumen de pacientes y permitan una distribución equitativa de la carga de trabajo. Las políticas que promueven los descansos, la programación rotativa y la dotación de personal de respaldo durante las pandemias u otras crisis pueden aliviar la fatiga y mejorar la retención. Los programas de capacitación también pueden mejorar la adaptabilidad del personal en todas las unidades, reduciendo la presión sobre el personal de enfermería de las UCI. La comunicación eficaz del liderazgo y la visibilidad son fundamentales en tiempos de crisis. Los directores de enfermería deben adoptar enfoques de gestión participativos que validen las experiencias de las enfermeras, fomenten la retroalimentación e involucren al personal en la formulación de políticas. Las instituciones también deben garantizar una comunicación transparente sobre la disponibilidad de EPP, los protocolos de seguridad y las directrices en evolución para reducir la incertidumbre y el estrés moral.

Los hospitales deben tratar el bienestar de las enfermeras de la UCI como una prioridad organizativa equivalente a la seguridad de los pacientes. El establecimiento de comités de bienestar, programas de reconocimiento para los trabajadores de primera línea y sistemas de recompensa por el trabajo en equipo y la práctica ética puede fomentar la moral y reforzar la identidad profesional. Además, la integración de métricas de salud mental y comentarios del personal en los marcos institucionales de mejora de la calidad puede garantizar la sostenibilidad y la rendición de cuentas. Al incorporar estas medidas en las estructuras institucionales, las organizaciones de asistencia sanitaria no solo pueden mitigar las cargas psicológicas y éticas descritas en este estudio, sino también reforzar la resiliencia de la plantilla, la retención y la calidad de la atención durante futuras emergencias de salud pública.

Las experiencias de las enfermeras de UCI turcas durante la pandemia de COVID-19 coinciden con los hallazgos globales, pero también reflejan características contextuales únicas arraigadas en la infraestructura sanitaria y los valores culturales de Turquía. Al igual que en estudios realizados en España, Irán e Italia, las enfermeras turcas informaron de cargas de trabajo excesivas, escasez de EPP, agotamiento emocional y angustia moral relacionados con la falta de personal y la rápida evolución de los protocolos de tratamiento^(3,6,17,18,22)^. En todos estos contextos, las enfermeras de la UCI demostraron un fuerte compromiso profesional y capacidad de adaptación a pesar del apoyo sistémico inadecuado. Sin embargo, la realidad turca también presenta dimensiones distintas que merecen atención. La rápida expansión de la capacidad de las UCI durante la pandemia en Turquía generó un aumento de la demanda de personal que no siempre se vio compensado por el apoyo de recursos humanos. Esto provocó un aumento de la carga de trabajo, la prolongación de la duración de los cuidados y tensiones éticas más frecuentes. A diferencia de algunos países, donde se establecieron sistemas estructurados de apoyo psicológico y de debriefing institucional en las primeras fases de la pandemia^(3,21)^, las enfermeras de las UCI turcas a menudo dependían de la solidaridad informal entre colegas y de mecanismos personales de afrontamiento. Además, el contexto cultural colectivista, caracterizado por un fuerte sentido del deber y la responsabilidad moral, pareció reforzar el compromiso ético de las enfermeras y su tendencia a continuar con la atención a pesar del riesgo personal. Este sentido de la obligación moral es paralelo a los resultados de estudios realizados en otros contextos^(6,19)^, en los que las normas de responsabilidad social y profesional moldearon de manera similar las respuestas de las enfermeras a la atención en situaciones de crisis. Al situar los hallazgos turcos en el panorama mundial, el estudio demuestra tanto la universalidad como la especificidad contextual de las experiencias de las enfermeras de las UCI durante la COVID-19. Los retos comunes a los distintos sistemas sanitarios ponen de relieve la necesidad de una colaboración internacional para desarrollar políticas institucionales basadas en la evidencia, en particular para reforzar los sistemas de apoyo psicológico, garantizar una dotación de personal adecuada y proteger la integridad ética y física de las enfermeras durante las emergencias sanitarias. El caso turco subraya que, incluso en países de ingresos medios con respuestas sanitarias rápidamente movilizadas, las limitaciones estructurales pueden obstaculizar la sostenibilidad de la atención integral. Por lo tanto, las lecciones aprendidas de Turquía pueden servir de base para estrategias regionales y globales destinadas a mejorar la resiliencia de los cuidados críticos.

Dado que los participantes trabajaban principalmente en las provincias occidentales y noroccidentales de Turquía, la generalización de los resultados es limitada. Además de la representación geográfica limitada de los participantes, la mayoría de los cuales trabajaban en el oeste y noroeste de Turquía. Este estudio se enfrentó a limitaciones metodológicas asociadas al uso de entrevistas en línea. La recolección de datos a través de la plataforma Zoom Cloud Meeting, aunque necesaria durante las restricciones pandémicas, puede haber influido en la profundidad y la espontaneidad de las respuestas de los participantes. El formato virtual puede haber reducido la sensación de conexión interpersonal, limitado las oportunidades para establecer una buena relación y restringido la capacidad de los investigadores para observar señales no verbales, como expresiones faciales, postura y matices emocionales, que son valiosos en la investigación fenomenológica. Aunque las descripciones verbales fueron ricas y reflexivas, la ausencia de una observación no verbal completa puede haber afectado a la interpretación de la intensidad emocional o los patrones de comunicación sutiles. Además, el hecho de basarse en experiencias autoinformadas introduce la posibilidad de un sesgo de recuerdo o un sesgo de deseabilidad social, ya que los participantes pueden haber moderado sus respuestas para ajustarse a las normas profesionales.

### Implicaciones para la Práctica Clínica y el Desarrollo de Políticas

El estudio hace hincapié en que los factores físicos, psicológicos y ambientales socavan colectivamente la calidad de la atención y el proceso de enfermería holístico, lo que supone un riesgo tanto para la seguridad de las enfermeras como para la de los pacientes. Subraya que proporcionar un apoyo institucional adecuado, incluyendo personal suficiente, servicios de salud mental y políticas organizativas de protección, es esencial para mantener una prestación de cuidados segura y holística en los entornos de cuidados intensivos. Los resultados también revelan que las enfermeras de la UCI experimentan un importante malestar moral y ético debido a las barreras sistémicas que limitan su capacidad para prestar una atención completa y equitativa. Especialmente durante crisis sanitarias como las pandemias, retos como la fatiga por el uso prolongado de EPP, la escasez de personal y el aplazamiento de las necesidades básicas ponen de relieve la necesidad de reformas institucionales y protocolos clínicos revisados para apoyar mejor a las enfermeras y salvaguardar los resultados de los pacientes.

### Revisar los Protocolos para Minimizar La Fatiga por el uso de EPP

Dado que el uso prolongado de EPP causaba dolores de cabeza, sudoración excesiva, deshidratación y retrasos en la satisfacción de las necesidades básicas, los hospitales deberían revisar los protocolos de control de infecciones existentes para equilibrar la seguridad con las necesidades fisiológicas. Se pueden introducir sistemas de rotación estructurados, en los que el personal alterne entre zonas de alta exposición y zonas de baja exposición, lo que permite intervalos programados para descansar, hidratarse y cambiar el EPP. El establecimiento de “zonas de recuperación” designadas cerca de las unidades de cuidados intensivos, donde las enfermeras puedan quitarse el EPP de forma segura, rehidratarse y refrescarse antes de volver a la atención, puede reducir sustancialmente la fatiga y mejorar la concentración. Además, la introducción de materiales mejorados para el EPP, ligeros, transpirables y ergonómicos, debe ser una prioridad en las políticas de adquisición para mejorar la comodidad sin comprometer la seguridad.

### Ajustar Los Horarios del Personal para Permitir Descansos Regulares e Hidratación

Los resultados del estudio demuestran que la escasez de personal y la prolongación de la duración de la atención a menudo provocaban agotamiento físico y disminución de la motivación. Las instituciones pueden abordar esta cuestión adoptando modelos de dotación de personal más flexibles y receptivos. Los sistemas de programación deben garantizar tiempo protegido para el descanso y la nutrición, incluso durante los picos de carga de pacientes. Los directivos pueden utilizar sistemas de seguimiento de la carga de trabajo o modelos de “dotación de personal basada en la gravedad” para distribuir las tareas de forma justa y en tiempo real, evitando el agotamiento y garantizando una prestación de cuidados equitativa. Además, la implementación de micro descansos breves pero frecuentes dentro de los turnos, como 20 minutos cada 4 horas, puede mejorar significativamente la concentración, reducir la fatiga y mantener la seguridad de los pacientes.

### Incorporar el Bienestar y el Apoyo a la Salud Mental en la Práctica Diaria

Más allá de la fatiga física, las enfermeras de este estudio informaron de tristeza, impotencia y agotamiento emocional. Para abordar esta situación, los hospitales deben integrar mecanismos de apoyo psicológico en las operaciones cotidianas, en lugar de tratarlos como intervenciones de emergencia. Las sesiones regulares de asesoramiento in situ, los círculos de apoyo entre compañeros y las reuniones informativas después de los turnos pueden proporcionar alivio emocional y fomentar la resiliencia. La incorporación de profesionales de la salud mental capacitados en los equipos de las UCI también puede ayudar a identificar los primeros signos de angustia y proporcionar apoyo oportuno.

### Mejorar el Trabajo en Equipo Interdisciplinario y la Capacidad de Respuesta del Liderazgo

Las experiencias de los participantes pusieron de relieve que los conflictos entre equipos y el apoyo inconsistente de la dirección intensificaban el estrés laboral. Fomentar la comunicación transparente, la toma de decisiones participativa y la responsabilidad mutua entre los miembros del equipo interdisciplinario puede reforzar la moral. Los líderes de enfermería deben realizar reuniones informativas periódicas para revisar los retos de la atención, recabar opiniones y comunicar claramente los cambios en las políticas de EPP, dotación de personal o flujo de trabajo.

### Integrar los Comentarios en la Mejora Continua de la Calidad

Los hospitales pueden institucionalizar mecanismos para que las enfermeras informen de forma anónima sobre sus preocupaciones relacionadas con la fatiga, la incomodidad de los EPP o los retos éticos. Los comentarios recolectados del personal de primera línea pueden servir de base para ciclos de mejora continua, lo que dará lugar a protocolos más receptivos y a una mayor confianza en la organización. Al traducir estas ideas en políticas operativas, las instituciones sanitarias pueden pasar de una gestión reactiva de las crisis a un modelo más sostenible de atención integral y centrada en el ser humano. Reducir el esfuerzo físico, garantizar un descanso adecuado e incorporar el apoyo psicológico no son solo medidas para proteger a las enfermeras, sino que son pasos fundamentales para salvaguardar la seguridad de los pacientes y preservar la integridad de la práctica de los cuidados intensivos.

## CONCLUSIÓN

El concepto de “El viaje a la UCI durante la pandemia de COVID-19”, tal y como se plantea en este estudio, captura la transición de las enfermeras de la UCI desde experiencias negativas y angustiosas a momentos de felicidad y satisfacción profesional. Los resultados revelan que las enfermeras de la UCI de COVID-19 se enfrentaron a un amplio espectro de experiencias asistenciales, que abarcaban tanto aspectos negativos como positivos. Las experiencias asistenciales negativas suponen un riesgo tanto para la seguridad de las enfermeras como para la de los pacientes. Las experiencias positivas sirven como fuente de motivación y esperanza, y animan a las enfermeras a seguir prestando asistencia. Factores como el aumento de la carga de trabajo, la fatiga, la escasez de personal, la falta de equipo, el riesgo de infección, las molestias físicas y psicológicas derivadas del uso de EPP, el apoyo insuficiente del equipo y la incapacidad de proporcionar una atención integral pueden comprometer la seguridad y la calidad general de la atención. Abordar estas cuestiones es fundamental para salvaguardar el bienestar tanto de las enfermeras como de los pacientes. Proporcionar un apoyo adecuado a las enfermeras de la UCI puede empoderarlas para desarrollar e implementar de manera efectiva modelos de atención holística.

El estudio puso de relieve tanto las debilidades como las fortalezas del sistema sanitario basándose en las experiencias de atención. Las enfermeras desempeñan un papel fundamental en el cuidado de los pacientes críticos. La atención de enfermería se ha visto significativamente afectada por factores como el miedo, el aislamiento, la escasez de personal, la insuficiencia de EPP, etc., lo que ha supuesto un reto para mantener la humanización de la atención sanitaria. Los resultados sugieren que fomentar la seguridad de las enfermeras y los pacientes durante el proceso de atención y abordar los retos que se plantean en el entorno laboral puede influir positivamente en las prácticas de enfermería. Mejorar la seguridad de las enfermeras de la UCI no solo mejora la seguridad de los pacientes, sino que también contribuye a la prestación de una atención integral a un nivel óptimo. Además, se prevé que estas medidas mejoren la satisfacción de las enfermeras y refuercen su compromiso con la profesión.

## Data Availability

El conjunto de datos que respaldan las conclusiones de este estudio está disponible previa solicitud al autor correspondiente.
